# Modulation of pain sensitivity by *Ascl1*- and *Lhx6*-dependent GABAergic neuronal function in streptozotocin diabetic mice

**DOI:** 10.1016/j.ymthe.2024.12.039

**Published:** 2024-12-30

**Authors:** Sung-Min Hwang, Md. Mahbubur Rahman, Eun Jin Go, Jueun Roh, Rayoung Park, Sung-Gwon Lee, Minyeop Nahm, Temugin Berta, Yong Ho Kim, Chul-Kyu Park

**Affiliations:** 1 Gachon Pain Center and Department of Physiology, Gachon University College of Medicine, Incheon, Republic of Korea; 2 Bio-IT Foundry Center of Chonnam National University and FromDATA, Buk-Gu, Gwangju, South Korea; 3 Section of Genetics and Physiology, Laboratory of Molecular and Cellular Biology, National Institute of Diabetes and Digestive and Kidney Diseases (NIDDK), National Institutes of Health (NIH), Bethesda, MD, USA; 4 Dementia Research Group, Korea Brain Research Institute, Daegu, Republic of Korea; 5 Pain Research Center, Department of Anesthesiology, University of Cincinnati Medical Center, Cincinnati, OH, USA

**Keywords:** diabetic neuropathic pain, dorsal root ganglion, GABAergic neuron, transcription factor, RNA sequencing

## Abstract

Painful diabetic neuropathy commonly affects the peripheral nervous system in individuals with diabetes. However, the pathological processes and mechanisms underlying diabetic neuropathic pain remain unclear. We aimed to identify the overall profiles and screen for genes potentially involved in pain mechanisms using transcriptome analysis of the dorsal root ganglion of diabetic mice treated with streptozotocin (STZ). Using RNA sequencing, we identified differentially expressed genes between streptozotocin-treated diabetic mice and controls, focusing on altered GABAergic neuron-related genes and inflammatory pathways. Behavioral and molecular analyses revealed a marked reduction in GABAergic neuronal markers (GAD65, GAD67, VGAT) and increased pro-inflammatory cytokines (TNF-α, IL-1β, IL-6) in the diabetic group compared with controls. Intrathecal administration of lentiviral vectors expressing transcription factors *Ascl1* and *Lhx6* reversed pain hypersensitivity and restored normal expression of GABAergic genes and inflammatory mediators. Protein-protein interaction network analysis revealed five key proteins influenced by *Ascl1* and *Lhx6* treatment, including those in the JunD/FosB/C-fos signaling pathway. These findings suggest that *Ascl1* and *Lhx6* mitigate diabetic neuropathic pain by modulating GABAergic neuronal function, pro-inflammatory responses, and pain-related channels (TRPV1, Nav1.7). These results provide a basis for developing transcription factor-based therapies targeting GABAergic neurons for diabetic neuropathic pain relief.

## Graphical abstract

**Figure:**
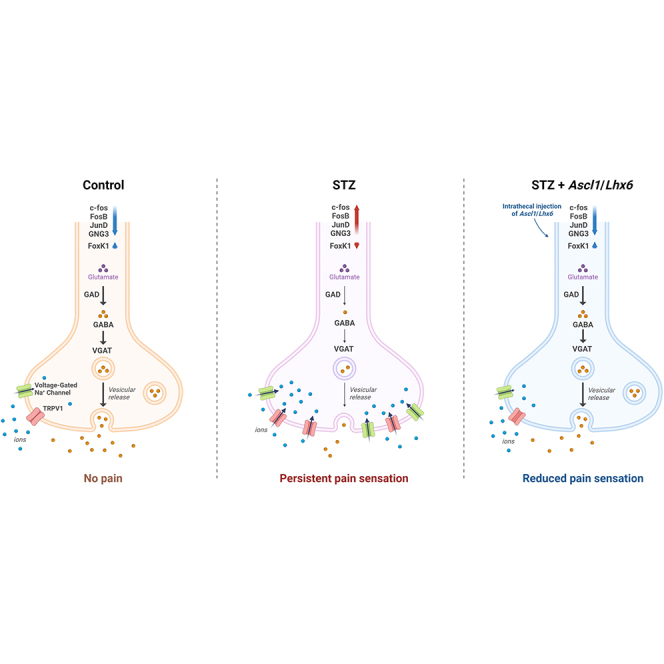

## Teaser

Park and colleagues investigate how the transcription factors Ascl1 and Lhx6 influence diabetic neuropathic pain by regulating GABAergic neuron function. Using transcriptome analysis and therapeutic gene delivery in diabetic mice, their study uncovers novel mechanisms and potential interventions for pain management, presenting promising therapeutic implications for diabetic neuropathy.

## Introduction

Diabetic neuropathic pain (DNP) is the most common chronic complication of untreated diabetes.^1^^,^^2^ Hyperexcitability (hypersensitivity) of the dorsal root ganglion (DRG) is a major cause of DNP.^3^^,^^4^ Numerous primary nociceptive clusters in the DRG play crucial roles in pain modulation, transduction, and transmission.^5^ Diabetic pathological conditions lead to various changes in gene expression related to the molecular, subcellular, and cellular levels, and network activity among cells in the peripheral nervous system (PNS).^6^ Peripheral nerve injury may also induce transcriptional reprogramming, changes in neuronal sensitivity, alterations in neuronal homeostasis, changes in gene expression, and a strong immune response in the DRG, resulting in altered pain hypersensitivity.^6^^,^^7^^,^^8^ Thus, DRG neurons are pivotal in pain development and the transition from acute to chronic (neuropathic) pain.^9^^,^^10^^,^^11^ However, the molecular and cellular mechanisms of DNP in the PNS have not been fully elucidated.

DRG neurons express multiple receptors for key neurotransmitters, including gamma-aminobutyric acid (GABA).^12^^,^^13^ GABAergic neurons produce GABA, which is the main inhibitory neuron in the mammalian central nervous system (CNS).^14^ Its inhibitory effects are mediated by anion-selective ionotropic GABA_A_ receptors and metabotropic GABA_B_ receptors.^15^^,^^16^ Activation of GABA_A_ receptors in the CNS neurons of most adults triggers chloride ion influx, resulting in membrane hyperpolarization and neuronal excitability suppression.^17^ In the PNS, DRG neurons express various proteins related to the functional GABAergic neuron required for GABA synthesis, transport, and release, and several GABA receptor subunits in inhibitory neurons.^18^ Recently, pharmacological, chemogenetic, or optogenetic stimulation of the GABAergic system within the DRG *in vivo* has been found to alleviate nociceptive, inflammatory, and neuropathic pain.^12^^,^^18^ Increasing evidence also indicates that functional and expression changes in GABAergic neurons are involved in the development and maintenance of various chronic pain conditions.^19^^,^^20^ However, the regulatory mechanisms of the effects of GABAergic neurons on DNP in the DRG remain insufficiently documented.

Many key transcription factors (TFs) regulate the fate acquisition process and specify different neuronal subtypes.^21^^,^^22^ DRG neurons express multiple neurotrophic receptors whose expression is controlled by TFs.^23^ Thus, an accurately selected cocktail of TFs can alter the fate of fully differentiated cells, enabling the achievement of functional neuronal fate.^24^ Recent studies indicate that peripheral injured models affect the expression levels of diverse TFs.^25^^,^^26^ However, TFs that control the GABAergic neuronal phenotype and function have not been studied in peripheral injury models.

Therefore, we aimed to perform a transcriptome analysis of the DRGs of streptozotocin (STZ) mice to identify the overall profiles and screen for genes potentially involved in pain mechanisms. Moreover, we aimed to investigate the functional roles of the TFs that regulate GABAergic neuronal function and pain-inducing signaling in modulating pain hypersensitivity in STZ diabetic mice.

## Results

### STZ-induced diabetic mice exhibited abnormal gene changes in related functional groups

**Figure 1:**
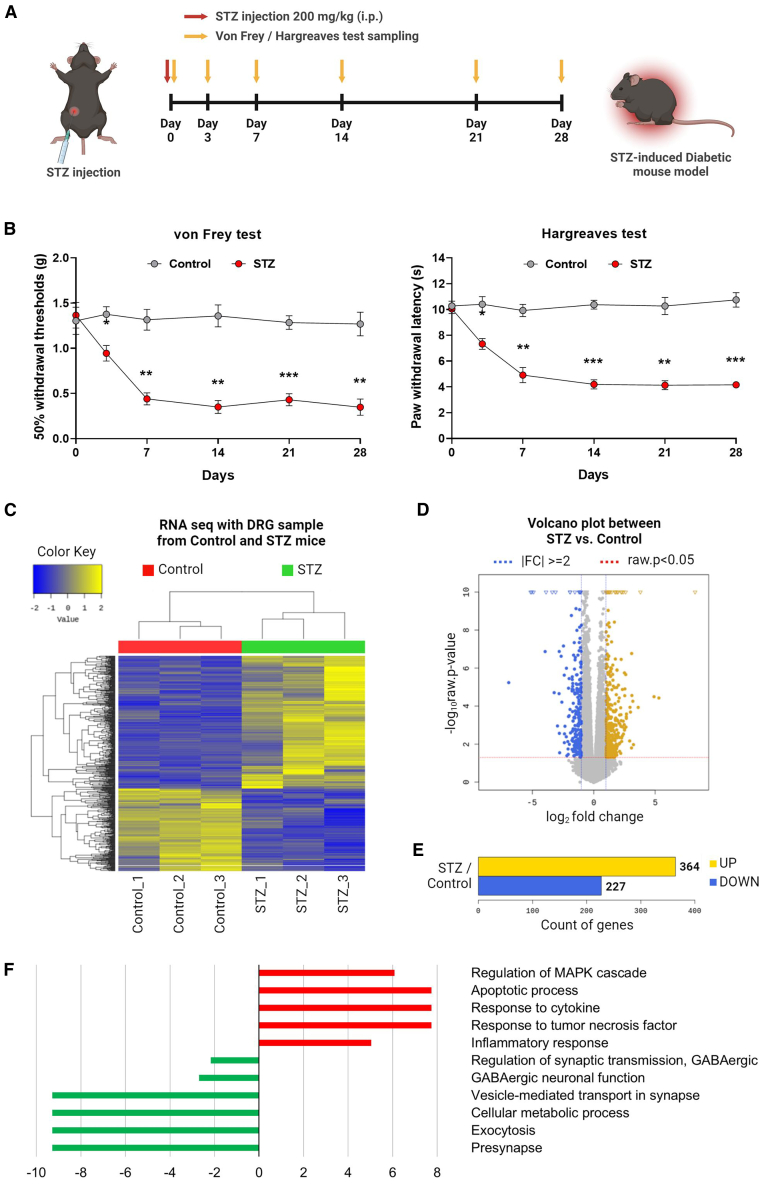
RNA sequencing transcriptional profiling and identification of abnormal pain-related gene expression in the DRG of diabetic mice (A) Timelines of the *in vivo* experimental design. (B) Mechanical and thermal tests in STZ-induced diabetic mice. (C) Hierarchical clustering analyses of DEGs. (D) Volcano plot of DEGs. (E) The number of upregulated and downregulated DEGs. (F) GO analysis of DEGs in the control versus STZ group. Data are reported as mean ± standard errors of the mean (*n* = 5). ∗*p* < 0.05, ∗∗∗*p* < 0.001 via the Bonferroni multiple comparisons test by two-way analysis of variance versus control. Data are shown for D0, D3, D7, D14, D21, and D28. STZ, streptozotocin.

An STZ-induced diabetic mouse model was established to investigate the regulatory mechanism of DNP. These mice were injected with STZ, and their pain responses were measured using von Frey and Hargreaves tests (Figure 1A). The results revealed that the STZ group exhibited a significant reduction in mechanical thresholds and thermal latency at 3 days, persisting until 28 days (Figure 1B). This result indicates the successful establishment of the STZ-induced diabetic mice as a model for DNP. Subsequent RNA sequencing (RNA-seq) was performed to compare the gene expression changes in the DRG between the STZ and control groups. Hierarchical clustering was used to compare the gene expression profiles and patterns of the two groups, as demonstrated by the separate clustering of the control and STZ groups (Figure 1C). In addition, the volcano plot indicated the number of differentially expressed genes (DEGs) (Figure 1D). The RNA-seq analysis identified 591 DEGs in the DRG of the STZ group, with 364 genes upregulated and 227 genes downregulated (Figure 1E). Based on the RNA-seq results, further Gene Ontology (GO) analysis was performed to explore the functional categories by linking groups likely associated with diabetic pain within the DRG. The analysis revealed that GABAergic neuronal function and related processes such as vesicle-mediated transport, exocytosis, presynapse, and cellular metabolic process were downregulated, whereas the MAPK cascade, apoptotic process, cytokine, and inflammatory response were upregulated (Figure 1F). These results suggest that abnormal changes in gene expression with similar functional groups may contribute to the induction of diabetic pain.

### STZ-induced diabetic mice exhibited downregulation of genes linked to GABAergic neuron function

**Figure 2:**
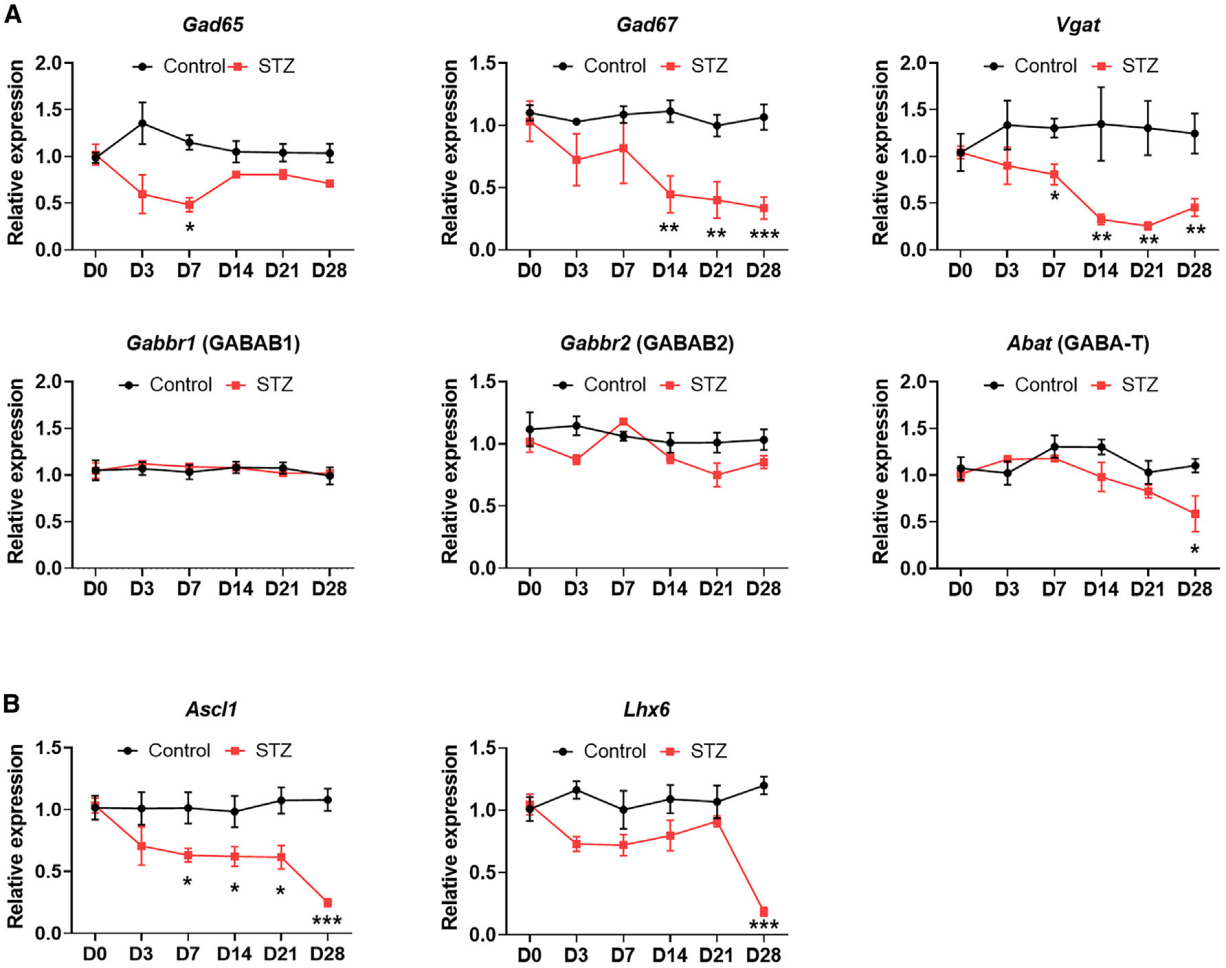
Downregulation of GABAergic neuron-specific genes and their transcription factors in the DRG with diabetes (A) Relative mRNA expression level of GABAergic neuron-specific genes. (B) Relative mRNA expression level of transcription factors in controlling the GABAergic neuron subtype identity. Data are reported as mean ± standard errors of the mean (*n* = 5). ∗*p* < 0.05, ∗∗*p* < 0.01, ∗∗∗*p* < 0.001, Dunnett’s multiple comparisons tests by one-way analysis of variance versus day 0. Data are shown for D0, D3, D7, D14, D21, and D28. STZ, streptozotocin.

We focused on abnormal GABA-related gene expression patterns to investigate GABAergic system dysfunction in the STZ-induced mouse model. Quantitative polymerase chain reaction (qPCR) analysis revealed that the genes regulating GABAergic neurons, including *Gad65*, *Gad67*, and *Vgat*, were significantly downregulated in the STZ group compared with the control group. In addition, the expression of GABA receptor subunit-related genes remained unchanged or slightly increased (Figures 2A and S1A). Subsequently, we assessed expression changes in TFs that affect GABAergic neuron development or function. Among the TFs examined, only the expression levels of achaete-scute family bHLH TF 1 (*Ascl1*) and LIM homeobox 6 (*Lhx6*) were significantly decreased in the DRG of STZ-induced diabetic mice on day 28, whereas other TFs such as distal-less homeobox 1 (*Dlx1*), *Dlx2*, *Dlx5*, *NKx2.1*, and *NKx2.2* showed no significant changes (Figures 2B and S1B). These findings indicate a downregulation in the expression of genes involved in the GABAergic neuron and specific TFs controlling GABAergic neuron function (i.e., *Ascl1* and *Lhx6*) in the STZ group.

### Pain hypersensitivity is alleviated by the synergistic effects of *Ascl1* and *Lhx6* through transgene expression in diabetic neuropathy

**Figure 3:**
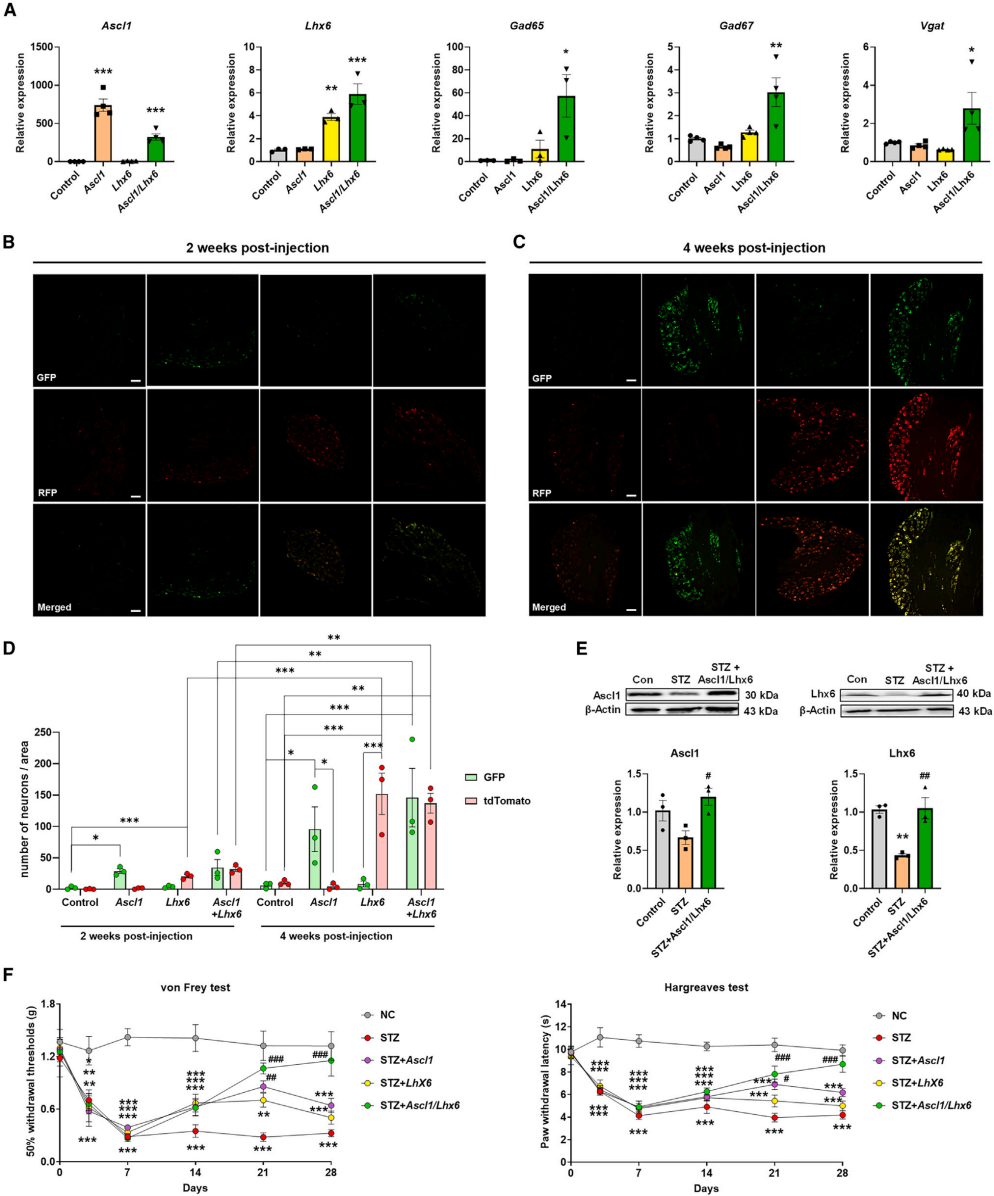
Synergistic effects of *Ascl1* and *Lhx6* through transgene expression alleviate mechanical allodynia and thermal hyperalgesia in diabetic neuropathy (A) Relative mRNA expression levels of GABAergic neuron-specific genes after treatment with *Ascl1* and *Lhx6* (*n* = 4). (B and C) Representative images showing GFP (green) and dTomato (red) fluorescence in mouse DRG tissues 2 weeks post-injection (B) and 4 weeks post-injection (C) of lentiviral vectors: empty vector (control), Lenti-hSyn-mAscl-P2A-EGFP (*Ascl1*), Lenti-hSyn-mLhx6-P2A-dTomato (*Lhx6*), and co-injection of Lenti-hSyn-mAscl-P2A-EGFP and Lenti-hSyn-mLhx6-P2A-dTomato (*Ascl1+Lhx6*). Scale bars, 50 μm. (D) Quantitative analysis of the number of EGFP- and tdTomato-positive cells per unit area in DRG tissues (*n* = 3). These data demonstrate the successful expression of *Ascl1* and *Lhx6* in DRG tissues and highlight the increased expression efficiency with time. (E) Relative protein expression levels of Ascl1 and Lhx6 in the three groups; β-actin was used as an internal control (*n* = 3). (F) Mechanical and thermal tests, using the von Frey test and Hargreaves test, respectively, in STZ-induced diabetic mice demonstrating the inhibitory effects of *Ascl1* and *Lhx6* on pain sensitivity (*n* = 5). Data are reported as mean ± standard error of the mean. ∗*p* < 0.05, ∗∗*p* < 0.01, ∗∗∗*p* < 0.001, Tukey’s multiple comparisons tests by two-way analysis of variance versus the naive group; #*p* < 0.05, ##*p* < 0.01, ###*p* < 0.001, versus the STZ diabetic control group. The data of non-significant points are not marked. STZ, streptozotocin.

Analysis of the results led us to hypothesize that increasing the expression levels of Ascl1 and Lhx6 could restore GABAergic gene expression and potentially alleviate pain in the STZ-induced diabetic mouse model. Thus, lentiviruses expressing Ascl1 and Lhx6 were used to individually or simultaneously infect cultured primary DRG neurons to identify changes in the expression of specific genes associated with GABAergic function. The qPCR data showed that the expression levels of *Gad65*, *Gad67*, and *Vgat* were slightly increased when *Ascl1* or *Lhx6* was expressed alone. However, their expression levels were significantly higher when *Ascl1* and *Lhx6* were co-expressed (Figure 3A). To evaluate the distribution and duration of transgene expression, DRG tissues were further analyzed at 2 and 4 weeks post-injection. Representative images (Figures 3B and 3C) illustrate green fluorescent protein (GFP) and tdTomato fluorescence in DRG tissues, demonstrating robust and sustained expression of Ascl1 and Lhx6. The corresponding quantitative analysis revealed a progressive increase in the number of GFP- and tdTomato-positive cells per unit area over time, highlighting the efficient and stable transduction achieved by the lentiviral vectors (Figure 3D). Furthermore, the protein expression of the two TFs was decreased in the STZ group, and the decrease was counteracted by expressing these two genes via the lentivirus (Figure 3E).

Based on these findings, we investigated their ability to modulate pain induction in the STZ group after intrathecal injection of a lentivirus expressing *Ascl1* and *Lhx6*. An increasing tendency of the paw withdrawal threshold and paw withdrawal latency were observed in the *Ascl1*- and *Lhx6* alone-treated groups until 21 days, and then decreased at 28 days, indicating that individual treatment with *Ascl1* or *Lhx6* is not sufficient to ameliorate the pain. However, a significant reduction in the mechanical thresholds and thermal latency in the STZ group was reversed to control levels by the expression of *Ascl1* and *Lhx6* (Figure 3F), suggesting that the combined expression of *Ascl1* and *Lhx6* may synergistically enhance GABAergic neuron function *in vitro* and *in vivo*. The behavioral test results at 14 days (2 weeks post-injection) did not indicate a significant decrease in the analgesic effect; the fluorescence results also indicated a limited number of GFP- and tdTomato-positive cells in DRG tissues (Figure 3B). However, as the number of GFP- and tdTomato-positive cells significantly increased by 4 weeks (Figure 3C), the behavioral test results corresponded to such findings, showing a marked improvement in pain relief (Figure 3F). This progression highlights the time-dependent efficiency of transgene expression in achieving analgesic effects.

Interestingly, the analgesic effect observed in the group treated with the combined expression of *Ascl1* and *Lhx6* was sustained until 42 days, whereas the effects in the *Ascl1*- and *Lhx6* alone-treated groups declined to levels similar to those observed at 28 days (Figure S2A). In addition, no signs of toxicity were detected among diabetic mice when comparing the diabetic control and TF-treated groups (Figure S2B; Table S1). Similarly, no significant changes in pain sensitivity, body weight, blood glucose levels, or food and water intake were observed in normal mice following the injection of Ascl1 and Lhx6, either separately or in combination (Figure S3; Table S2).

In addition, co-localization with NeuN, a neuronal marker, confirmed that GFP (reporter for *Ascl1*) and tdTomato (reporter for *Lhx6*) expression was restricted to neurons and not observed in non-neuronal cells (Figure S4). These findings suggest that the changes in the protein expression of Ascl1 and Lhx6 may play a crucial role in pain modulation in DNP.

### *Ascl1* and *Lhx6* restore the altered gene expression levels of GABAergic neurons, pro-inflammatory, TRPV1, and Nav1.7

**Figure 4:**
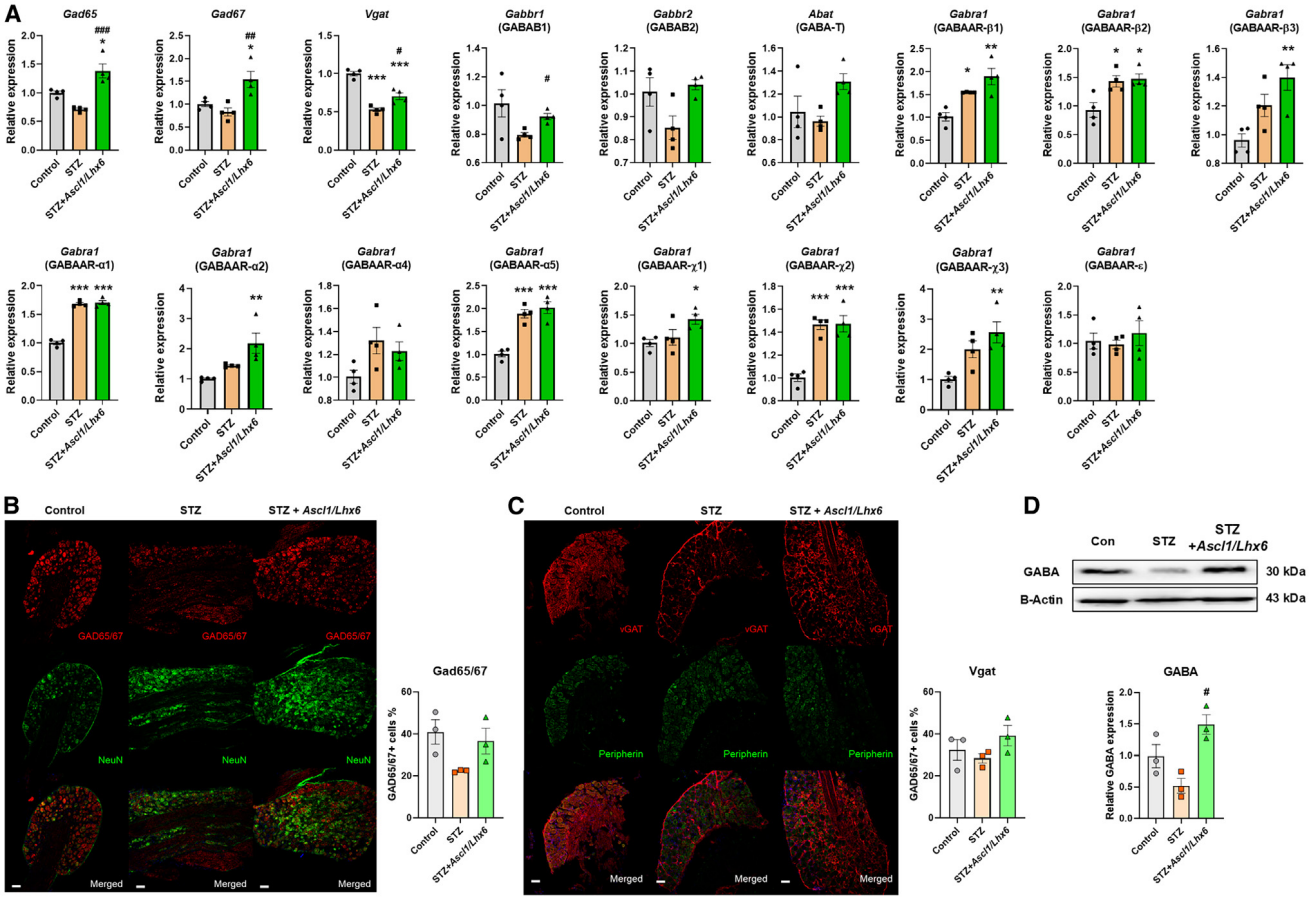
Regulatory effects of *Ascl1* and *Lhx6* on GABAergic neuron-specific genes in the DRG of diabetic mice (A) Relative mRNA expression level of GABA-related genes in the three groups. (B and C) Relative protein expression level of GABAergic neuron-specific protein, Gad65/67, and Vgat in three groups. (D) Relative protein expression level of GABA in the three groups; β-actin was used as an internal control. Data are reported as mean ± standard error of the mean (*n* = 3). ∗*p* < 0.05, ∗∗∗*p* < 0.001, Tukey’s multiple comparisons tests by two-way analysis of variance versus the naive group; #*p* < 0.05, ##*p* < 0.01, ###*p* < 0.001, versus the STZ diabetic control group. The data of non-significant points are not marked. STZ, streptozotocin.

We compared the expression levels of GABAergic genes among the three groups (control, STZ, and *Ascl1/Lhx6*-treated STZ) to investigate the specific genes involved in pain relief following the overexpression of *Ascl1* and *Lhx6*. The results showed that the *Ascl1/Lhx6*-treated STZ group had a significantly increased expression level of a gene related to the GABAergic system (*Gabbr1* for GABAB1 gene), which was reduced in the STZ group. However, this treatment did not affect the expression of GABA receptor subunit-related genes, specifically GABAARs (Figure 4A). Further analysis identified proteins associated with GABAergic neuronal function. Immunohistochemistry (IHC) results revealed a similar pattern of protein expression changes for Gad65, Gad67, and Vgat, which are involved in GABA release (Figures 4B and 4C). In addition, changes in GABA protein expression were consistently confirmed through western blotting (WB) (Figure 4D), suggesting that these TFs primarily enhance the function of GABAergic neurons in DRG neurons.

**Figure 5:**
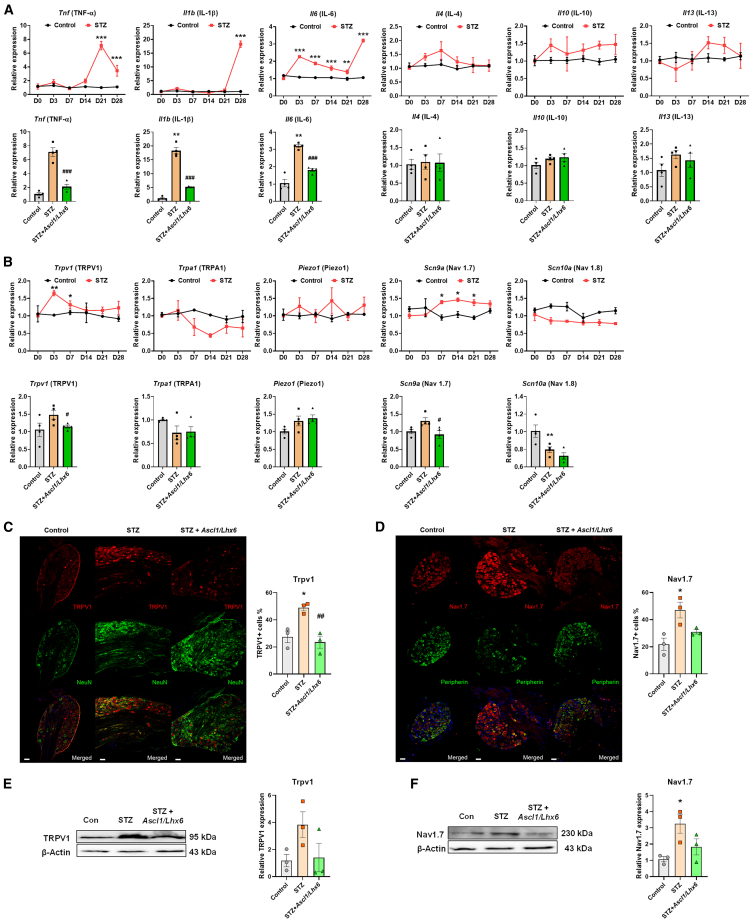
Regulatory function of *Ascl1* and *Lhx6* on GABAergic neuron-specific genes, pro-inflammatory and anti-inflammatory cytokines and pain-related channels in the DRG of diabetic mice (A) Relative mRNA expression level of pro- and anti-inflammatory cytokine-related factors in the three groups. (B) Relative mRNA expression level of pain-related channels in the three groups. (C and D) Relative protein expression levels of TRPV1 and Nav1.7 in the three groups. (E and F) Relative protein expression level of GABA in the three groups; β-actin was used as an internal control. Data are reported as mean ± standard error of the mean (*n* = 3). ∗*p* < 0.05, ∗∗∗*p* < 0.001, Tukey’s multiple comparisons tests by two-way analysis of variance versus the naive group; #*p* < 0.05, ###*p* < 0.001, versus the STZ diabetic control group. STZ, streptozotocin.

Next, we examined the expression levels of pro-inflammatory cytokines and pain-induced channels/receptors in the three groups. The gene expression levels of pro-inflammatory cytokines, such as tumor necrosis factor (*TNF*), interleukin-β (*IL-β*), and *IL-6*, were significantly increased in the STZ group, and these elevated levels were inhibited by the overexpression of *Ascl1* and *Lhx6* (Figure 5A). However, the expression levels of anti-inflammatory cytokines *IL-4*, *IL-10*, and *IL-13* did not show significant changes in the STZ group. Furthermore, the expression levels of pain-induced channels, such as *Trpv1* and *Scn9a* (for Nav1.7 gene), displayed a significant increase in the STZ group, and these elevated levels were reduced in the *Ascl1/Lhx6*-treated STZ group (Figure 5B). Protein expression levels related to TRPV1 and Nav1.7 were also examined in the three groups. The IHC results showed a similar pattern of protein expression changes for Trpv1 and Nav1.7 (Figures 5C and 5D). These changes in Trpv1 and Nav1.7 protein expression were consistently confirmed through WB (Figures 5E and 5F). These findings suggest that *Ascl1* and *Lhx6* regulate the expression levels of various genes involved in pathological conditions, including pain induction and inflammation, in DRG neurons.

### *Ascl1* and *Lhx6* regulate the physiological inhibitory function related to pain sensitivity

**Figure 6:**
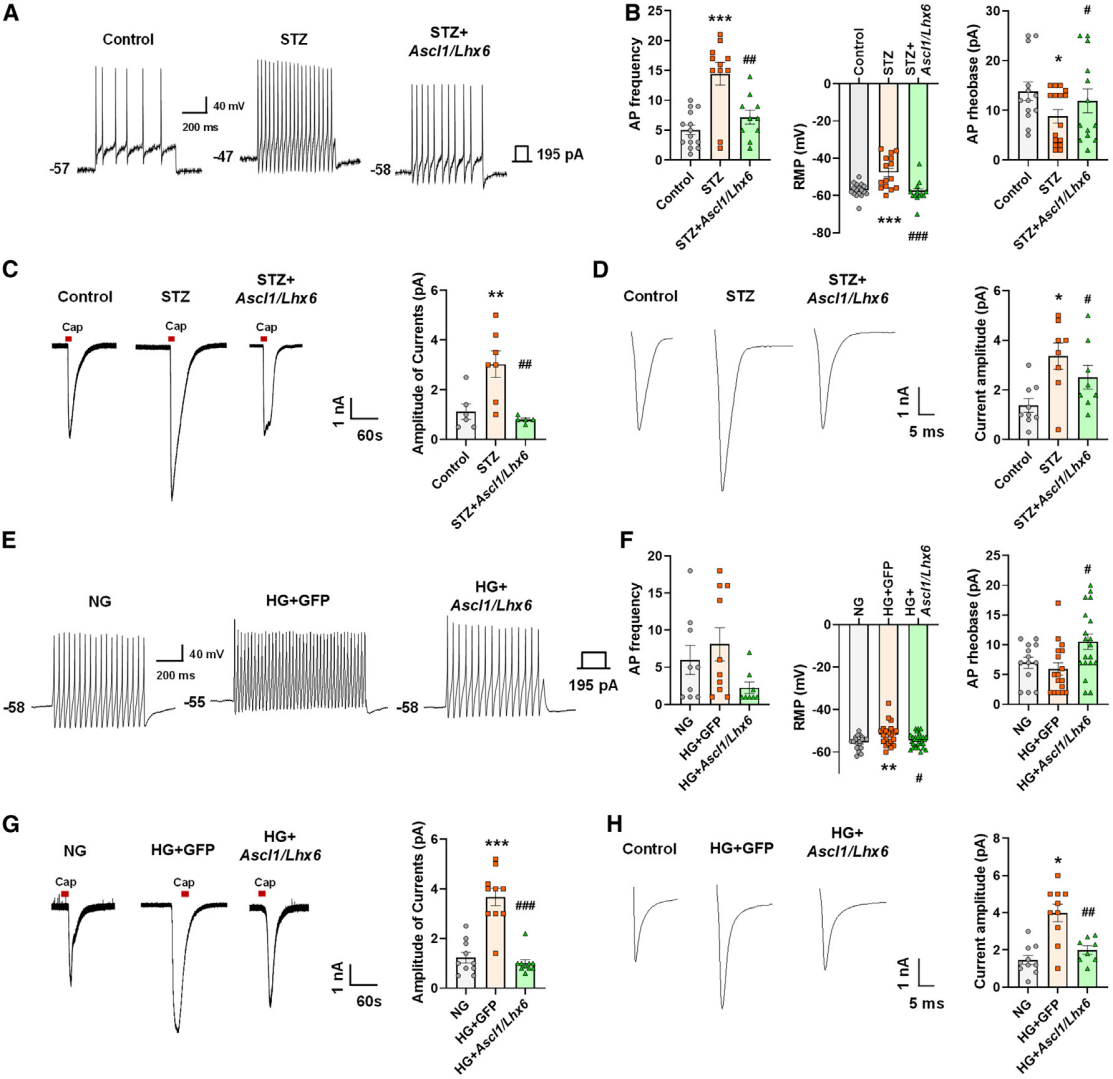
Regulatory effects of *Ascl1* and *Lhx6* on neuronal hyperexcitability, TRPV1, and Navs in the DRG of diabetic mice (A) Representative example of the AP frequency, RMP, and minimum current (rheobase) in the three groups *in vivo*. (B) Comparison of the AP frequency, RMP, and minimum current (rheobase) in the three groups *in vivo*. (C) Representative example (left) and comparison (right) of TRPV1 activation in the DRG neurons in the three groups *in vivo*. (D) Representative example (left) and comparison (right) of the transient Na^+^ current in small-sized DRG neurons in the three groups *in vivo*. (E) Representative example of the AP frequency, RMP, and minimum current (rheobase) in the three groups *in vitro*. (F) Comparison of the AP frequency, RMP, and minimum current (rheobase) in the three groups *in vitro*. (G) Representative example (left) and comparison (right) of TRPV1 activation in the DRG neurons in the three groups *in vitro*. (H) Representative example (left) and comparison (right) of transient Na^+^ current activation in small-sized DRG neurons in the three groups *in vitro*. Data are reported as mean ± standard error of the mean. ∗*p* < 0.05, ∗∗*p* < 0.01, ∗∗∗*p* < 0.001, Tukey’s multiple comparisons tests by two-way analysis of variance versus the naive group; #*p* < 0.05, ##*p* < 0.01, ###*p* < 0.001, versus the STZ diabetic control group. STZ, streptozotocin.

We examined the excitability of DRG neurons using whole-cell patch-clamp electrophysiology across the three groups to uncover the mechanism by which *Ascl1* and *Lhx6* reduce pain hypersensitivity in STZ-induced diabetic mice. The results indicated that the STZ group exhibited an increase in the action potential (AP) frequency, a decrease in the resting membrane potential (RMP), and an increase in AP rheobase. *Ascl1* and *Lhx6* reversed these physiological functional changes to the control level (Figures 6A and 6B). We recorded capsaicin-induced inward current in dissociated small-sized DRG neurons from the three groups to determine if TRPV1 activation influences pain induction in STZ mice. In the DRG of the STZ group, TRPV1 activation was enhanced, whereas it was reduced in the *Ascl1/Lhx6*-treated STZ group (Figure 6C). Similarly, we measured the function of voltage-gated sodium channels (Navs), including Nav1.7, in small-sized DRG neurons. The DRG of the STZ group exhibited an increase in the transient sodium ion (Na^+^) current. The *Ascl1/Lhx6* treatment in the STZ group significantly decreased the level of transient Na^*+*^ current (Figure 6D).

Furthermore, we observed that DRG neurons exhibited hyperexcitable properties, including enhanced transient Na^*+*^ currents (Nav1.7) and capsaicin-induced inward currents, after 24 h of incubation in high glucose (HG) (45 mM of glucose) medium. Cells were either left untreated (NG) (normal level of glucose), treated with EGFP lentivirus in HG (HG *+* GFP), or treated with *Ascl1/Lhx6* in HG. The HG *+* GFP group showed a significant increase in AP frequency, a reduction in RMP, and an increase in AP rheobase compared with the NG group. However, the application of TFs reversed these changes to levels comparable with the NG group (Figures 6E and 6F). Capsaicin-induced inward currents, which were augmented in the HG *+* GFP group, were significantly reduced in the HG group treated with *Ascl1/Lhx6* TFs (Figure 6G). Similarly, the HG-GFP group exhibited an increase in the transient Na^*+*^ current, which was substantially reduced following treatment with *Ascl1/Lhx6* in the HG group (Figure 6H). These findings suggest that the physiological activation of the hyperexcitable condition, including TRPV1 and the transient Na^*+*^ current in the STZ group, was mitigated by *Ascl1/Lhx6* treatment, potentially contributing to the inhibition of pain induction.

### *Ascl1* and *Lhx6* reverse the expression changes in JunD/FosB/C-fos signaling in STZ-induced diabetic mice

**Figure 7:**
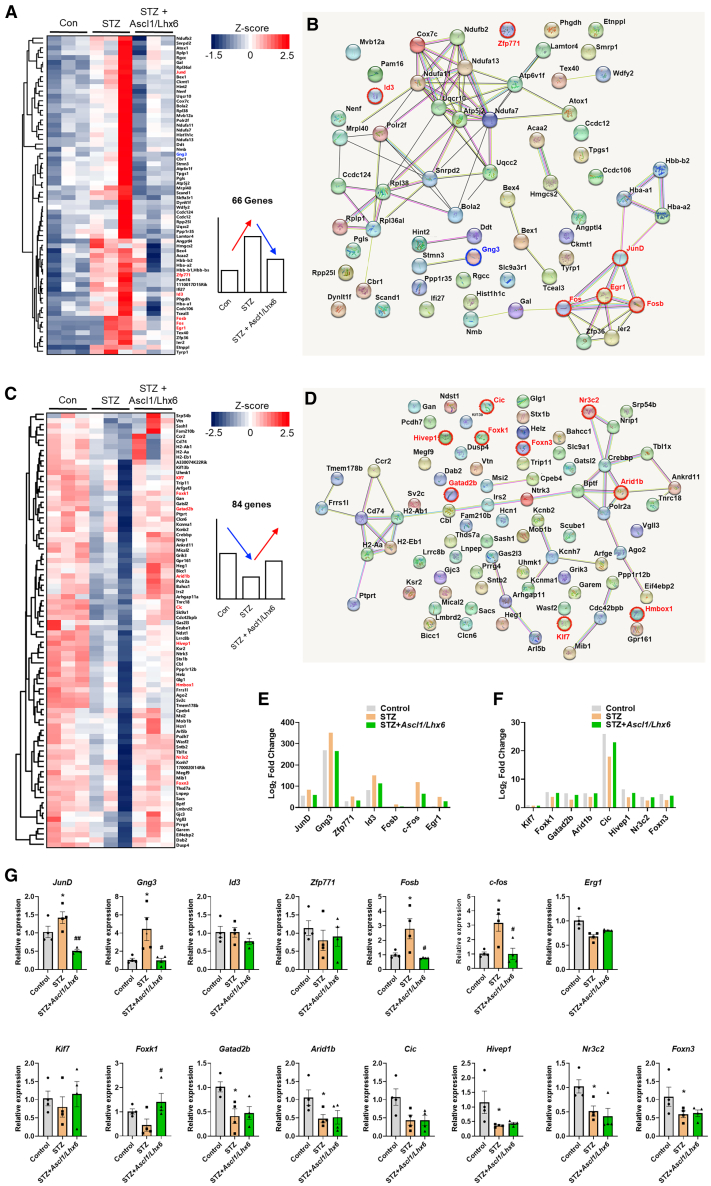
Identification of candidate genes influenced by *Ascl1* and *Lhx6* in the DRG of diabetic mice (A–D) Hierarchical clustering heatmap of DEGs and the network generated by STRING database analysis in the three groups. (E and F) Of the genes identified in (A), 15 are significantly expressed. (G) Relative mRNA expression levels of 15 candidate genes in the 3 groups. Data are reported as mean ± standard error of the mean (*n* = 3). ∗*p* < 0.05, ∗∗*p* < 0.01, Tukey’s multiple comparisons tests by two-way analysis of variance versus the naive group; #*p* < 0.05, ##*p* < 0.01, versus the STZ diabetic control group. STZ, streptozotocin.

**Figure 8:**
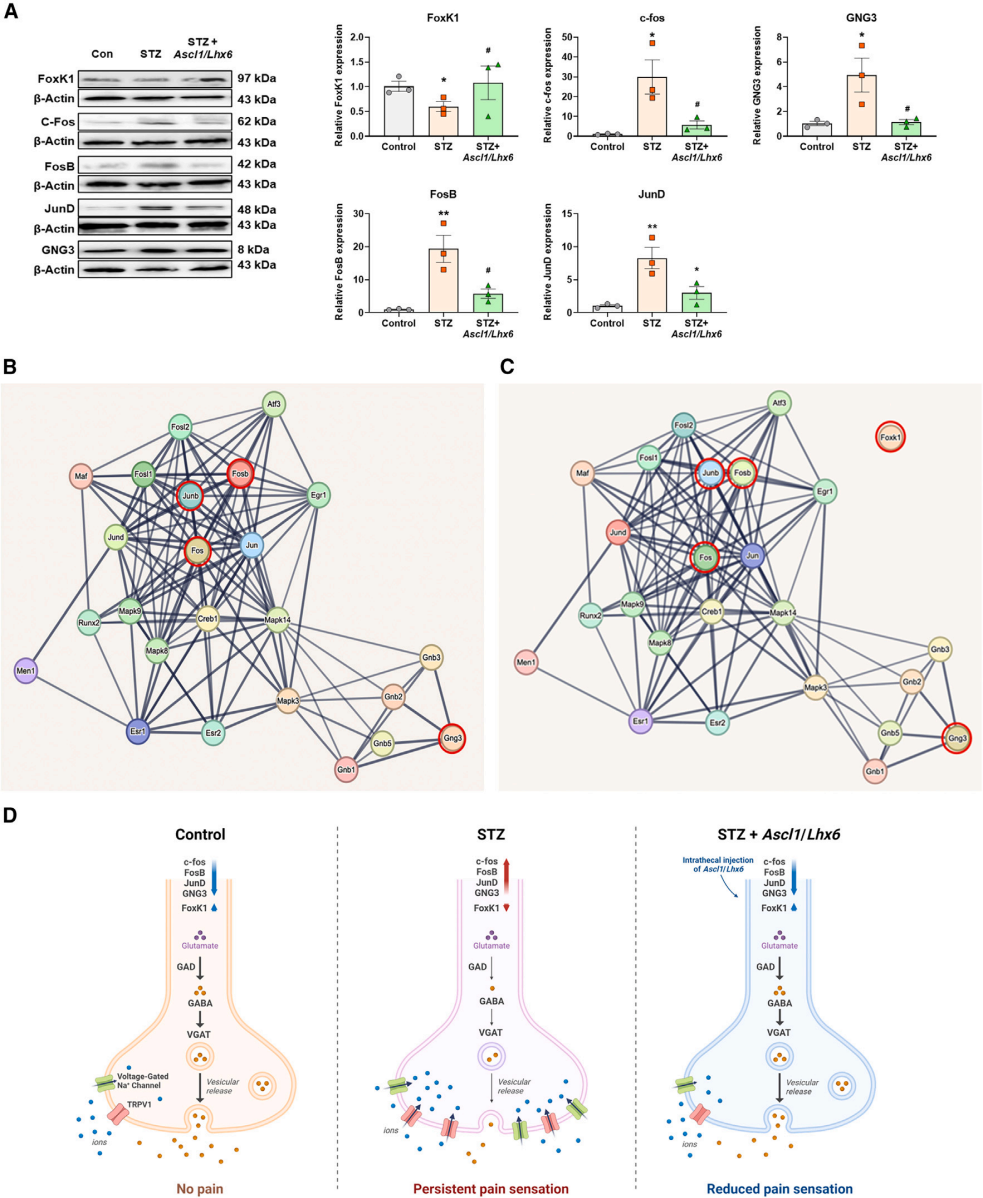
Identification of five proteins in the DRG of diabetic mice (A) Expression changes in FoxK1, C-Fos, FosB, JunD, and GNG3 via immunoblot analysis in the three groups; β-actin was used as an internal control (*n* = 3). (B and C) Network generated by STRING database analysis using the DEGs from (A). (D) Proposed schematic of the molecular mechanisms underlying pain regulation in the three conditions (no pain, persistent pain sensation, and reduced pain sensation) through the overexpression of Ascl1/Lhx6. Data are reported as mean ± standard error of the mean. ∗*p* < 0.05, ∗∗*p* < 0.01, Tukey’s multiple comparisons tests by two-way analysis of variance versus the naive group; #*p* < 0.05, ##*p* < 0.01, versus the STZ diabetic control group. STZ, streptozotocin.

We have demonstrated that *Ascl1* and *Lhx6* can alleviate pain sensitivity in STZ-induced diabetic mice. However, the specific mechanism by which they regulate pain remains unclear. Consequently, we explored candidate genes influenced by GABAergic neuronal function following *Ascl1* and *Lhx6* activation in the three experimental groups using hierarchical clustering and protein-protein interaction (PPI) network analyses. Our findings revealed 66 upregulated and 84 downregulated genes in the STZ group (Figures 7A–7D). Of these, we identified seven genes (including *JunD*, *FosB*, *C-fos*, guanine nucleotide-binding protein G[I]/G[S]/G[O] subunit gamma-3 [*Gng3*], *zfp771*, *Id3*, and *Erg1*) and eight genes (including *Kif7*, *Foxk1*, *Gatad2b*, *Arid1b*, *Cic*, *Hivep1*, *Nr3c2*, and *Foxn3*) that exhibited significant changes between the STZ and *Ascl1/Lhx6*-treated STZ groups (Figures 7E and 7F). We conducted real-time PCR using the identified candidate genes to validate the RNA-seq analysis results. RT-qPCR confirmed that four genes (including *JunD*, *Fosb*, *C-fos*, and *Gng3*) were upregulated in the STZ group, and that their activation was reduced in the *Ascl1/Lhx6*-treated STZ group (Figure 7G). Conversely, Foxk1 was downregulated in the STZ group, and its expression was increased in the *Ascl1/Lhx6*-treated STZ group (Figure 7G). In addition, WB analysis confirmed that JunD, FosB, C-fos, Gng3, and FoxK1 exhibited similar expression patterns compared with the RT-qPCR results (Figure 8A).

To investigate the relationship between these five proteins (including JunD, FosB, C-fos, Gng3, and FoxK1) and their interacting proteins, we constructed a PPI network for 4 or 5 protein groups using STRING. The analysis showed extensive PPI between JunD, FosB, C-fos, and Gng3 and 20 other proteins (Figure 8B), as well as between JunD, FosB, C-fos, Gng3, and FoxK1 and another set of 20 proteins (Figure 8C). Using co-immunoprecipitation (coIP), we examined the PPIs specifically among JunD, FosB, and c-Fos. Interestingly, JunD exhibited increased binding with c-Fos and FosB in the STZ group compared with the control, whereas this binding was consistently reduced in the *Ascl1/Lhx6*-treated STZ group (Figure S5). Although Foxn3 and Gng3 were part of the PPI network, we could not confirm their direct interactions with JunD/FosB/C-fos under the tested conditions. These findings suggest that the interactions among JunD, FosB, and c-Fos are enhanced under diabetic conditions and attenuated by *Ascl1* and *Lhx6*.

## Discussion

Painful diabetic neuropathy (PDN) is a widely known and common complication of diabetic neuropathy, representing the most prevalent form of neuropathic pain associated with diabetes.^27^^,^^28^ Understanding the mechanism underlying PDN in the peripheral system is critical for preventing its progression and discovering better treatments for various aberrant pain sensations.^29^^,^^30^ Following painful injuries caused by diabetes, primary sensory neurons exhibit maladaptive and hyperexcitable behavior, contributing to peripheral sensitization, which plays a crucial role in the initiation and maintenance of chronic pain.^31^ In addition, changes in the pathologic plasticity and various modalities of DRG neurons appear to be hallmarks of chronic pain.^31^ In this study, we analyzed the DRG through RNA-seq to identify differentially regulated gene neurons in STZ-induced diabetic mice compared with the control group. We discovered that GABAergic neuron-specific genes and TFs controlling GABAergic neuronal subtype identity are downregulated in the DRG neurons of STZ-induced diabetic mice. Therefore, we explored the potential of TFs to improve overall GABAergic neuron function and evaluated how effective such an intervention could be in alleviating pain. Our findings demonstrated that *Ascl1* and *Lhx6* can significantly attenuate thermal and mechanical pain sensitivity and regulate GABAergic neuronal function, pro-inflammatory conditions, and the physiological functions of TRPV1 and Nav7 in DRG neurons, which may play important roles in DNP.

GABAergic neurons play a crucial role in modulating pain transmission by continuously releasing GABA to decrease the excitability of lamina I-II output neurons under normal physiological conditions.^32^ Growing evidence suggests that inhibiting GABAergic neurons plays an important role in the manifestation of neuropathic and inflammatory pain conditions.^32^ Peripheral nerve injuries can lead to a decrease or dysfunction of GABAergic function in the dorsal horn, significantly impacting pain processing and modulation.^32^^,^^33^ The GABAergic system involves GABA synthesis from glutamate through the action of two isoforms of glutamate decarboxylase (GAD), namely, GAD65 and GAD67.^12^^,^^34^^,^^35^ GABA, packed into synaptic vesicles by vesicular GABA transporters (VGAT), is released into the synaptic cleft. It then binds to GABA receptors on postsynaptic neurons, leading to neuronal activity inhibition.^36^ GABA receptors are classified into the ionotropic GABA_A_ receptors and metabotropic GABA_B_ receptors.^37^ There are 19 GABA_A_ receptor subunits (including α1-6, β1-3, γ1-3, δ, ε, θ, π, and ρ1-3) and two principal GABA_B_ receptor subunits (GABA_B1_ and GABA_B2_).^17^^,^^34^^,^^38^ GABA transporters, which consist of four GABA transporters (GATs 1–4), remove GABA from the postsynaptic cleft.^34^ Thus, the hypofunction of the GABA receptor is involved in the pathogenesis of allodynia.^39^ Therefore, our results showing increased expression of GABAergic neuron-related genes in the DRG of STZ diabetic mice may serve as a promising treatment strategy for inhibiting pain.

STZ-induced diabetic rodents exhibit increased expression of Navs.^40^^,^^41^ Injury to DRG neurons results in a lower threshold for activation in response to thermal and mechanical stimuli, and this hyperexcitability has been linked to the accumulation of Navs in these neurons.^42^ DRG neurons express two types of Nav channels: TTX sensitivity (Nav1.6 and Nav1.7) and TTX resistant (Nav1.8 and Nav1.9).^43^ Nav1.7 and 1.9 are primarily expressed in small DRG neurons and have been directly linked to pain-related hypersensitivity.^41^^,^^44^ In our study, we measured the activity of Navs, including Nav1.7, in small DRG neurons and observed a decrease in the expression of *Ascl1* and *Lhx6* in the presence of increased levels of primarily Nav1.7 current in the STZ group. These findings provide evidence for the involvement of primarily Nav1.7 in pain activation and regulation, as observed in our results. TRPV1 is a nonselective cation channel expressed in primary sensory neurons^45^ and a polymodal detector that is sensitive to various stimuli, including capsaicin, proton, and heat.^46^ Under pathological conditions, the function of TRPV1 in DRG neurons and spinal dorsal horn is altered, leading to changes in mechanical and heat sensitivity.^47^^,^^48^^,^^49^ Thus, inhibiting TRPV1 function alleviates thermal and mechanical pain sensitivity resulting from inflammation or nerve injury models.^47^^,^^48^^,^^49^ Our findings demonstrated that *Ascl1* and *Lhx6* can alleviate the pain sensation induced by STZ, providing evidence of the involvement of TRPV1 and Nav1.7 in the regulation of thermal and mechanical pain sensitivity and chronic pain.

Increasing evidence suggests that GABA plays a crucial role in pain regulation in DRG.^18^^,^^19^ However, whether TFs that control the identity of the GABAergic neuronal subtype in the sensory neurons of the DRG play a role in the changes in pain-associated genes in pathological conditions and whether they have a functional role in diabetes-induced pain remain unknown. TFs serve as master switches, contributing to cell specification and differentiation to determine the final fate of neurons.^50^^,^^51^ TFs mediating the fate of GABAergic neurons include Ascl1, Lhx6, Dlx, and NK2 homeobox 1 and 2 (NKx2.1 and NKx2.2).^50^^,^^52^^,^^53^ Ascl1 plays an important role in developing telencephalon and inducing cortical interneurons.^54^^,^^55^ Lhx6, a gene subtype of the LIM homeodomain family, plays an essential role in the migration of GABAergic interneuron from medial ganglionic eminence to the cortex in mice.^56^ DLX1, 2, 5, and 6, which are expressed in the CNS during brain development, contribute to promoting GABAergic function.^57^^,^^58^ NKX2.1/2 promotes the specification and differentiation of GABAergic and cholinergic cell fate in various tissues.^59^ Particularly, Ascl1 serves as a master regulator and plays a pivotal role in neuronal development.^60^^,^^61^^,^^62^^,^^63^ In contrast, Lhx6 alone does not significantly affect neuronal phenotype changes but enhances efficiency and neuronal quality.^60^ In this study, we observed that co-treatment with *Ascl1* and *Lhx6* led to a greater increase in the expression of GABAergic neuron-specific genes than with *Ascl1* treatment alone (Figure S6).

Trans-differentiation, also known as direct cell reprogramming, is a process by which various cell types, including fully differentiated ones, are converted into specific functional neurons without undergoing an intermediate state.^55^^,^^64^ Numerous studies have proposed various protocols for acquiring cell fate.^24^^,^^65^ Therefore, a direct approach to specifying neuronal subtypes could involve overexpressing specific TFs.^22^^,^^24^ TFs play multiple roles in cell fate decisions and reprogramming cells from one cell type into another.^22^^,^^24^^,^^64^ Several key TFs that regulate neuronal identity have been identified and are now utilized for specific neuronal subtypes.^65^^,^^66^^,^^67^ Several studies have focused on deriving GABAergic neurons from human induced pluripotent stem cells and embryonic stem cells using various TFs.^53^^,^^68^ The neurotransmitter phenotype of the neurons is primarily determined early in development.^69^ However, neuronal neurotransmitter phenotypes exhibit greater plasticity than what might be expected from the rigid wiring scheme observed in adult neurons. For instance, excitation and inhibition (i.e., the two fundamental interactions between neurons) utilize glutamate and GABA as the major neurotransmitters for excitatory and inhibitory signals, respectively.^70^^,^^71^ Furthermore, studies have shown that hippocampal mossy fiber synapses can release excitatory glutamate and inhibitory GABA neurotransmitters.^69^ Therefore, if it was possible to experimentally induce differentiated neurons to switch their neurotransmitter phenotype to another, such as the GABAergic phenotype, it would be valuable to have a sufficient supply of neurons with this neurotransmitter phenotype. However, this possibility has not yet been explored.

Glutamate, as an excitatory neurotransmitter, is pivotal in both healthy and disease states, including neuropathic and inflammatory pain, and is essential for the glutamatergic pathways within the peripheral and CNSs.^72^ It is generated in the cytoplasm and sequestered into synaptic vesicles by vesicular glutamate transporters (VGLUTs), where it is then released to enable excitatory neurotransmission.^73^ The disruption of the balance between excitatory glutamatergic and inhibitory GABAergic neuronal activities is a well-documented contributor to the development of peripheral neuropathic pain. In the STZ-induced model, a notable dip in GABAergic activity was noted alongside a maintained or marginally heightened activity in glutamatergic neurons, which appeared to be modulated by distinct neurotransmitter profiles. To target pain modulation, we aimed to modulate neurotransmitter phenotype switching. This involved upregulating GABAergic neurons through the induction of TFs such as Ascl1 and Lhx6. Our investigation into the influence of TFs on glutamatergic neurons revealed minimal alterations in the expression of *vGlut1-2* and their related TFs *Tbr1-2*, *NeuroD1*, and *NeuroG2* in primary DRG neurons. Nonetheless, an increase in *TBR2* and *NeuroD1* was observed in STZ-treated mice, and the *Ascl1/Lhx6* treatment effectively diminished *NeuroD1* levels. The TFs played a crucial role in the recovery of GABAergic functionality and in lowering the levels of particular genes linked to glutamatergic activity under STZ treatment, indicating a recalibration toward equilibrium facilitated by the GABAergic neurotransmitter switch (Figure S7). Thus, our study suggests the potential for inducing a new neurotransmitter phenotype that enhances GABAergic neuronal functions in DRG neurons through the application of *Ascl1* and *Lhx6*.

We discovered that 15 genes out of 150 DEGs were significantly upregulated or downregulated in the 3 groups. Subsequently, 5 of these genes, which showed significant changes, were validated via real-time PCR and WB in the STZ and *Ascl1/Lhx6*-treated STZ group, respectively. Particularly, activating protein-1 can regulate the inflammatory response; protein-1 consists of 18 dimeric complexes from four DNA-binding proteins families: Jun (v-Jun, c-Jun, JunB, and JunD), Fos (v-Fos, c-Fos, FosB, Fral, and Fra2), ATF/cyclic AMP-responsive element-binding (ATF2, ATF3/LRF1, B-ATF, JDP1, and JDP2), and Maf (c-Maf, MafB, MafA, MafG/F/K, and Nrl).^74^^,^^75^^,^^76^ JunD is expressed in various cell types in response to physiological and pathophysiological stimuli and interacts with ATF3, MEN1, DNA damage-inducible transcript 3, and BRCA1.^77^^,^^78^^,^^79^^,^^80^^,^^81^ Fos proteins, including c-Fos, FosB, and deltaFosB, are a family of TFs.^82^^,^^83^ FosB, an immediate-early response gene, serves as an indicator of neuronal activation and is involved in the excitotoxic activation of microglia.^84^ c-Fos is a marker of neuronal activation following noxious stimulation and tissue injury.^85^ Gng3 affects regulators of neuronal excitability.^86^ Foxn3 is involved in numerous cellular and physiological processes, including cell proliferation, aging, obesity, and cancer.^87^ Foxn3 inhibits ubiquitin-mediated IkB-α degradation and NF-κB transcriptional activity.^88^ Thus, the PPI network analysis identified interactions involving 20 proteins centered around the JunD/FosB/C-fos signaling pathway. Among these, Gng3 was confirmed to interact with these core proteins, while Foxn3 exhibited distinct interaction patterns and may not be directly involved in these complexes. We discovered that Foxn3 exhibits a different interaction pattern with four of these proteins. In addition, we observed changes in the interaction of JunD with FosB and C-fos between the STZ group and *Ascl1/Lhx6*-treated STZ group, consistent with the coIP results. However, despite their inclusion in the PPI network, we could not confirm direct interactions between Foxn3 or Gng3 and JunD/FosB/C-fos. This highlights a potential limitation in the experimental conditions used and suggests that the mechanism by which JunD/FosB/C-fos signaling regulates pain, particularly through other proteins such as Foxn3 and Gng3, requires further investigation.

Determining how and where to target substances for treating PDN are complex challenges. Some studies have explored pain control by intrathecally injecting pain-relieving substances into the spinal cord to examine how pain regulation is influenced by various cells within the CNS^89^^,^^90^^,^^91^ or by directly injecting into the DRG within the PNS to focus on DRG-mediated pain regulation.^91^^,^^92^ In addition, the mechanism of pain regulation through viral vector-mediated drug function can vary depending on the promoter type used in the vector. For example, a virus with a cytomegalovirus promoter can express substances in various cells, including nerve cells, whereas a virus with a synapsin promoter may specifically target nerve cells, potentially leading to differences in the pain regulation process.^93^^,^^94^ Our previous study confirmed the pain-relieving effects in the spinal cord by using a lentivirus-containing cytomegalovirus promoter to deliver two TFs via intrathecal injection.^95^ Consequently, we aimed to elucidate pain mechanisms within the DRG using a lentivirus-containing synapsin promoter for the intrathecal injection of *Ascl1* and *Lhx6*. A sequence of transcriptional and molecular shifts within neuronal cells impacted pain perception across three experimental conditions: the control group, the STZ-induced diabetic model, and the Ascl1/Lhx6-treated STZ group. The control group maintained a state of no pain due to the harmonious expression of TFs (*c-Fos*, *FosB*, *JunD*, *GNG3*, and *FoxK1*), with neurotransmitters and ion channels, including voltage-gated sodium channels and TRPV1 receptors, functioning normally. In the STZ-induced model, there was an increase in pain sensation. This is reflected by the heightened expression of c-Fos and FosB, decreased levels of JunD and FoxK1, increased activity of voltage-gated sodium channels, and upregulation of TRPV1 receptors. Such changes result in diminished GABA synthesis, as shown by the reduced presence of GAD and VGAT. In the *Ascl1/Lhx6*-treated STZ group, however, there was a notable reduction in pain sensation. This improvement correlated with the restoration of TFs to normal levels, which in turn rebalanced GABA production, and also led to an adjustment in the expression and function of the voltage-gated sodium channels and TRPV1 receptors. These adjustments indicate potential reversal of the neuropathic pain phenotypes induced by STZ.

Overall, our study elucidated the roles of *Ascl1* and *Lhx6* in mitigating neuropathic pain and in the functional regulation of GABAergic neurons, pro-inflammatory signaling, TRPV1, and Nav channels in the DRG neurons of diabetic mice. In addition, we found that JunD/FosB/C-fos signaling could modulate pain sensitivity in the DRG of the *Ascl1/Lhx6*-treated STZ group. These findings support a strategy for managing DNP through the functional mechanisms of *Ascl1* and *Lhx6*.

## Materials and methods

### Reagents

STZ, glucose, and capsaicin were purchased from Sigma (St. Louis, MO). The stock solutions were made with deionized water or 100% ethanol. All stock solutions were stored at −20°C.

### Animals

The Institutional Animal Care and Use Committee of the College of Medicine at Gachon University reviewed and approved all surgical and experimental procedures (approval no.: LCDI-2019-0072). Animal experiments were performed according to the guidelines of the International Association for the Study of Pain. Adult male C57BL/6N mice, 6 weeks old, were purchased from Orientbio (Sungnam, South Korea) and acclimated to a 12-h light/12-h dark cycle for at least 1 week before the experiments.

### Lentivirus vector generation

We designed three lentivirus constructs: Lenti-hSyn-mAscl-P2A-EGFP, Lenti-hSyn-mLhx6-P2A-tdTomato, and Lenti-hSyn-EGFP. Each vector was packaged by the KIST virus facility. The respective titers were 5.29 × 10^10^, 5.37 × 10^10^, and 2.9 × 10^11^ vg/mL.

### Diabetes induction and blood glucose level tests

Diabetes mellitus was induced using a single intraperitoneal injection of STZ (200 mg/kg; Sigma-Aldrich) that was freshly dissolved in citrate buffer (pH 4.5).^95^ Mice in the control group were injected with an equivalent volume of the vehicle. Hyperglycemia (blood glucose concentration of >300 mg/dL [16.7 mmol/L]) confirmed diabetes mellitus 3 and 7 days after STZ injection using an Accu-Chek Compact Plus glucose meter (Roche Diagnostics, Meylan, France). STZ-injected animals not exhibiting hyperglycemia 3 days after injection were excluded from the study. Blood glucose was measured at 0, 3, 7, 14, 21, 28, 35, and 42 days.

### Evaluation of toxicity

The mice were carefully examined visually for any signs of adverse or toxic effects prior to the commencement of the experiment, during the treatment period, and just prior to sacrifice. The visual examination included checking for mortality, behavioral changes (e.g., weakness, aggressiveness, paraplegia, uni- or quadriplegia, tremor, jaw stiffness, dysphasia, salivation, and food or water refusal), defecation (diarrhea or loose feces), noisy breathing and colonic convulsion, dyspnea, discharge from the eyes and ears, and voice changes. TFs *Ascl1* and *Lhx6* were administered either individually or in combination to two distinct batches of mice: normal mice (naive control group) and diabetic mice (STZ group). The amount of food and water consumption was recorded every 3 days.

### Determination of diabetes-induced neuropathic pain

To measure the paw withdrawal threshold in response to mechanical stimuli, the animals were first placed in a plexiglass chamber with a 4 × 3 mm wire mesh grid floor and allowed to acclimatize for 30 min. After the acclimatization period, calibrated von Frey filaments (g) (NC12775-99, North Coast Medical, Morgan Hill, CA) were applied perpendicularly to the plantar surface of the right hind paw with sufficient force to bend the filament for 6 s or until the paw was withdrawn. Rapid withdrawal or paw flinching was interpreted as a positive response. If there was a response, the next lower force filament was applied. If there was no response, the next higher force filament was applied. The behavioral test was performed before STZ or vehicle injection (baseline day 0) and then on days 3, 7, 14, 21, and 28 following STZ injection. The 50% paw withdrawal threshold was calculated as described previously.^96^ Similarly, additional diabetic mouse treated batch were performed at 0, 3, 7, 14, 21, 28, 35, and 42 days points, while naive batch groups vehicle injection (baseline day 0) and then on days 3, 7, 14, 21, and 28 following vehicle injection. Thermal hyperalgesia was measured by recording the paw withdrawal latency using a Hargreaves radiant heat apparatus. The cutoff value was set to 20 s to prevent tissue damage.

A stable diabetic state was achieved through a single intravenous STZ injection (200 mg/kg) 3 and 7 days (>300 mg/dL) before the single intrathecal lentiviral administration on day 7. Virus preparations were concentrated to a titer of 7 × 10^9^ vg/mL via tangential crossflow ultrafiltration. The methods and time points of paw withdrawal threshold measurement were the same as described previously.^96^^,^^97^

### Mouse DRG neuron cultures

DRGs were harvested from mice (6–9 weeks old) and were incubated with collagenase A (0.2 mg/mL, Roche, Basel, Switzerland)/dispase-II (2.4 units/mL, Roche) at 37°C for 90 min. Cells were mechanically dissociated with gentle pipetting. DRG cells were plated on poly-D-lysine-coated coverslips and cultured in neurobasal culture medium with 10% fetal bovine serum (Gibco, Waltham, MA), 2% B27 supplement (Invitrogen, Carlsbad, CA), and 1% penicillin/streptomycin for 1 day before patch-clamp recordings.

### *In vitro* induction of the diabetic peripheral neuropathy model

DRG cultures were divided into three groups to establish an *in vitro* model of diabetic peripheral neuropathy. The first group served as the control and received a standard glucose concentration (normal glucose, NG group with 25 mM of glucose). The second group, treated with EGFP lentivirus, was given an HG concentration (HG *+* GFP group with 45 mM of glucose). The third group, treated with *Ascl1* and *Lhx6* lentivirus, was also given HG (HG *+ Ascl1/Lhx6* group). The DRG neurons from the three groups were cultured with or without *Ascl1* and *Lhx6* lentivirus treatment for 4 days. On the following day, each experiment was conducted.

### RNA purification, cDNA synthesis, and RNA-seq analysis

Total RNA was isolated from the DRG using TRIzol (Thermo Fisher Scientific, 15596026). Subsequently, cDNA was synthesized using a reverse transcription kit (MMLV, Invitrogen, 28025013) according to the manufacturer’s instructions. After quality assessment and quantification using the Bioanalyzer 2100 (Agilent Technologies, Palo Alto, CA), 1 μg of the total RNA from each sample was processed using the Illumina TruSeq Stranded mRNA Library Prep Kit (Illumina, San Diego, CA) according to the manufacturer’s instructions. The resulting cDNA libraries were sequenced on the Illumina platform and approximately 685 million, 101 bp paired-end reads were generated. The raw sequence data have been deposited in the NCBI-SRA database under the following accession numbers: SRR28519807–SRR28519812 within PRJNA1094985. The raw sequenced reads underwent preprocessing using Trimmomatic (v.0.36) to eliminate low-quality sequences and adaptors (24695404). Subsequently, the trimmed reads were aligned to the mouse reference genome (mm10) employing HISAT2 (v.2.1.0) with default parameters (31375807). Quantification of known transcripts was conducted using the aligned reads obtained from the previous step. This quantification was performed using Cufflinks (v.2.2.1). Gene expression levels were normalized using fragments per kilobase of exon per million fragments mapped (FPKM) (20436464). Differential gene expression analysis was conducted utilizing Cuffdiff (v.2.1.1) (23222703). DEGs were identified based on an FPKM value greater than 1 in at least one sample and a false discovery rate less than 0.05. These DEGs were considered significant and subjected to further analysis. The mouse genome reference sequence and annotations were obtained from the University of California, Santa Cruz (UCSC) browser (https://genome.uscs.edu). GO and pathway enrichment analysis of the DEGs was performed using the DAVID (v.6.8) functional annotation tool (https://david.ncifcrf.gov) (19131956) and Kyoto Encyclopedia of Genes and Genomes pathway database (https://www.genome.jp/kegg) (27899662), respectively. PPI networks of DEGs were analyzed using the STRING (v.12.0) database (https://string-db.org) with the following parameters: confidence score >0.7 and primary interactions <20 or 50 (36370105).

### Real-time PCR

Gene expression analysis was performed using real-time qPCR. Total RNA was extracted from DRG samples using TRIzol (Thermo Fisher Scientific, 15596026), and cDNA was synthesized using a reverse transcription kit (MMLV, Invitrogen, 28025013) according to the manufacturer’s instructions. qPCR was performed using the SYBR Green PCR Kit (Bio-Rad, 170-8882AP). Table S3 lists all the designed primer sequences. PCR was performed at 95°C for 30 s, followed by 40 cycles at 60°C for 30 s, at 72°C for 30 s, at 95°C for 10 s, and at 65°C for 5 s. Data were normalized to the mRNA level of glyceraldehyde 3-phosphate dehydrogenase, which was used as an internal control.

### WB

DRG samples were homogenized in an ice-cold tissue protein extraction reagent (NP-40, EBA-1049, Elpis Biotech, Daejeon, Korea) supplemented with a 1% proteinase inhibitor mix. The protein concentration was measured using a bicinchoninic acid assay kit (Thermo Fisher Scientific, 23227), and samples were separated using sodium dodecyl sulfate-polyacrylamide gel electrophoresis and electro-transferred onto a polyvinylidene fluoride membrane (Millipore, ipvh00010). The blots were blocked with 5% non-fat (skim) milk in Tris-buffered saline with Tween (TBST) and incubated overnight with primary antibodies, including rabbit anti-Ascl1 (catalog no. PA5-77868, 1:500, Invitrogen), mouse anti-GABA (catalog no. MAB316, 1:500, Sigma-Aldrich), mouse anti-Lhx6 (catalog no. sc-271433, 1:200, Santa Cruz Biotechnology), mice anti-VR1 (catalog no. sc-398417, 1:1,000, Santa Cruz Biotechnology), rabbit-SCN9A (catalog no. ASC-008, 1:200, Alomone Labs), rabbit anti-c-Fos (catalog no. 4384, 1:1,000, Cell Signaling Technology), rabbit anti-FoxK1 (catalog no. 12025, 1:1,000, Cell Signaling Technology), rabbit anti-JunD (catalog no. 5000, 1:1,000, Cell Signaling Technology), rabbit anti-FosB (catalog no. 2251, 1:1,000, Cell Signaling Technology), and rabbit anti-GNG3 (catalog no. DF9559, 1:1,000, Affinity Bioscience). Each membrane was washed with TBST and incubated for 1 h with the secondary antibodies, anti-rabbit Ig-horseradish peroxidase (catalog no. 9910, 1:10,000, Cell Signaling Technology), and *m*-IgGκ BP-HRP (catalog no. sc-516102, 1:1,000, Santa Cruz Biotechnology). After washing with TBST, the immune complexes were detected via chemiluminescence (Beyotime Biotechnology, Shanghai, China) using a Pierce Western blotting kit. Quantitative densitometric analysis was performed using a UVP BioSpectrum multispectral imaging system (GE Healthcare Life Sciences, ImageQuant LAS 4000, San Diego, CA).

### co-IP

DRG samples were lysed in an ice-cold immunoprecipitation buffer containing Tris-HCl, NaCl, MgCl_2_, ethylene-diamine-tetraacetic acid (EDTA), Triton X-100, and glycerol, along with a protease inhibitor cocktail. The lysate was precleared with protein A/G PLUS-agarose and immunoprecipitated with specific rabbit antibodies (anti-c-Fos, anti-JunD, anti-FosB, anti-FoxK1, and anti-GNG3). Immunoprecipitates were collected and washed three times with a wash buffer (50 mM Tris-HCl [pH 7.4], 150 mM NaCl, 10 mM EDTA, and 1% NP-40).

### IHC

Mice from each group were anesthetized with isoflurane and perfused with phosphate-buffered saline (PBS), followed by 4% paraformaldehyde (PFA) in PBS. The L3-L5 DRGs were postfixed with 4% PFA overnight. Tissues were then embedded at the optimal cutting temperature after dehydration in 20% and 30% sucrose for 24 h, and sectioned into 14-μm-thick slices using a cryostat. The sections were blocked with 5% fetal bovine serum for 1 h at 25°C and incubated overnight at 4°C with the following primary antibodies: rabbit anti-GABA transporter (GAT-1) (catalog no. AGT-001, 1:250, Alomone Lab), rabbit anti-GAD65 + GAD67 (catalog no. ab183999, 1:250, Abcam), mouse anti-vGAT (catalog no. ab307448, 1:50, Abcam), mouse anti-VR1 (R-130) (catalog no. sc-398417, 1:100, Santa Cruz), guinea pig anti-Nav1.7 (catalog no. ASC-008, 1:200, Alomone Lab), mouse anti-NeuN (catalog no. MAB377, 1:200, Sigma-Aldrich), rabbit anti-peripherin antibody (catalog no. AB1530, 1:200, Sigma-Aldrich), GFP (catalog no. A-11122, 1:200, Invitrogen), and tdTomato (catalog no. A121690, 1:400, antibodies.com). The fluorescence of the sections was examined under an LSM700 confocal microscope.

### Patch-clamp recording

Whole-cell patch-clamp recordings were conducted at 25°C using MPC-200 manipulators (Sutter Instrument, Novato, CA) and a Multiclamp 700B amplifier (Molecular Devices, San Jose, CA). The patch pipettes were pulled from borosilicate capillaries (Chase Scientific Glass, Rockwood, TN), with a resistance of 2–5 MΩ, and series resistance was compensated (>80%). Data were low-pass filtered at 2 kHz and sampled at 10 kHz. Voltage-clamp recordings were performed at a holding potential of −70 mV. The pipette solution for voltage-clamp experiments contained: 122.5 mM K-gluconate, 12.5 mM KCl, 0.2 mM EGTA, 8 mM NaCl, 2 mM MgATP, 0.3 mM Na_3_GTP, and 10 mM HEPES adjusted to pH 7.3 with KOH. The bath solution contained: 140 mM NaCl, 5 mM KCl, 1 mM MgCl_2_, 10 mM HEPES, 10 mM glucose, and 2 mM EGTA. In the current-clamp mode, a current injection evoked the APs. The RMP was measured without a current injection. The step protocol (from 0 to 200 pA in 10 pA increments with a pulse duration of 500 ms) was applied to determine the rheobase. To record Na^+^ currents, the pipette solution contained: 13 mmol/L CsCl, 9 mmol/L NaCl, 1 mmol/L MgCl_2_, 10 mmol/L EGTA, 10 mmol/L HEPES, 0.5 mmol/L Na GTP, and 2 mmol/L NaATP adjusted to pH 7.4 with CsOH. The external solution for recording transient Na^+^ currents contained: 131 mmol/L NaCl, 10 mmol/L TEACl, 10 mmol/L CsCl, 1 mmol/L CaCl_2_, 2 mmol/L MgCl_2_, 0.3 mmol/L CdCl_2_, 3 mmol/L 4-aminopyridine, 10 mmol/L HEPES, and 10 mmol/L glucose adjusted to pH 7.4 with NaOH. TEA-Cl and CdCl_2_ were puffed extracellularly onto the neurons to block endogenous voltage-gated potassium and calcium channels. In voltage-clamp recording, a transient Na^+^ current was evoked by a test pulse to 0 mV from the holding potential −70 mV. The recording chamber (300 μL) was continuously perfused (3–4 mL/min) with a focal perfusion system. The perfusion exchange time was 1–2 s from merging manifold from the recording chamber.

### Statistical analysis

Statistical analyses were conducted using GraphPad Prism 8 (GraphPad Software, San Diego, CA). All data are presented as the mean ± standard error of the mean. Differences between groups were compared using the two-tailed unpaired t test for two groups, one-way analysis of variance (ANOVA) followed by Dunnett’s multiple comparison test for multiple groups, or two-way repeated measures ANOVA followed by the Bonferroni multiple comparison test for multiple groups and time courses. The statistical significance threshold was *p* < 0.05.

## Data and code availability

The data that support the findings of this study are available in this article and its supplemental material. Additional data are available from the corresponding authors upon reasonable request.

## Acknowledgments

This study was supported by grants from the 10.13039/501100003725National Research Foundation of Korea, South Korea (NRF- 2021R1I1A1A0105260011 to S.-M.H.) and the Bio & Medical Technology Development Program of the National Research Foundation of Korea, South Korea (NRF- 2021R1A5A2030333 to Y.H.K.). We also acknowledge the support of the KBRI Basic Research Program through the 10.13039/100018749Korea Brain Research Institute, South Korea, funded by the 10.13039/501100014188Ministry of Science and ICT (24-BR-02-04 to M.N.), and 10.13039/100000002US National Institutes of Health (NS113243 to T.B.).

## Author contributions

Conceptualization, S.-M.H. and Y.H.K.; methodology, S.-M.H. and R.P.; investigation, S.-M.H., M.M.R., E.J.G., J.R., and S.-G.L.; data analysis, S.-G.L. and R.P.; supervision, Y.H.K. and C.K.P.; writing – original draft, S-.M.H. and E.J.G.; writing – review & editing, S.-M.H., T.B., M.N., and C.K.P.; funding acquisition, S.-M.H., Y.H.K., M.N., and T.B.

## Declaration of interests

The authors declare no competing interests.

## Supplemental information

Document S1. Figures S1–S7 and Tables S1–S3

Document S2. Article plus supplemental information

## References

[1] Pop-Busui R., Ang L., Boulton A.J.M., Feldman E.L., Marcus R.L., Mizokami-Stout K., Singleton J.R., Ziegler D. (2022).

[2] Schreiber A.K., Nones C.F., Reis R.C., Chichorro J.G., Cunha J.M. (2015). Diabetic neuropathic pain: Physiopathology and treatment. World J. Diabetes.

[3] Chung J.M., Chung K. (2002). Importance of hyperexcitability of DRG neurons in neuropathic pain. Pain Pract..

[4] Reyes-Pardo H., Sanchez-Herrera D.P., Santillan M. (2022). On the effects of diabetes mellitus on the mechanical properties of DRG sensory neurons and their possible relation with diabetic neuropathy. Phys. Biol..

[5] Millan M.J. (2002). Descending control of pain. Prog. Neurobiol..

[6] Meacham K., Shepherd A., Mohapatra D.P., Haroutounian S. (2017). Neuropathic Pain: Central vs. Peripheral Mechanisms. Curr. Pain Headache Rep..

[7] Ydens E., Cauwels A., Asselbergh B., Goethals S., Peeraer L., Lornet G., Almeida-Souza L., Van Ginderachter J.A., Timmerman V., Janssens S. (2012). Acute injury in the peripheral nervous system triggers an alternative macrophage response. J. Neuroinflammation.

[8] Ellis A., Bennett D.L.H. (2013). Neuroinflammation and the generation of neuropathic pain. Br. J. Anaesth..

[9] Krames E.S. (2014). The role of the dorsal root ganglion in the development of neuropathic pain. Pain Med..

[10] Zhang J., Chen S.R., Chen H., Pan H.L. (2018). RE1-silencing transcription factor controls the acute-to-chronic neuropathic pain transition and Chrm2 receptor gene expression in primary sensory neurons. J. Biol. Chem..

[11] Sikandar S., Minett M.S., Millet Q., Santana-Varela S., Lau J., Wood J.N., Zhao J. (2018). Brain-derived neurotrophic factor derived from sensory neurons plays a critical role in chronic pain. Brain.

[12] Du X., Hao H., Yang Y., Huang S., Wang C., Gigout S., Ramli R., Li X., Jaworska E., Edwards I. (2017). Local GABAergic signaling within sensory ganglia controls peripheral nociceptive transmission. J. Clin. Invest..

[13] Yu L., Ding Y., Spencer A., Ma J., Lu R., Rudkin B.B., Yuan C. (2012). Dorsal root ganglion progenitors differentiate to gamma-aminobutyric acid- and choline acetyltransferase-positive neurons. Neural Regen. Res..

[14] Watanabe M., Maemura K., Kanbara K., Tamayama T., Hayasaki H. (2002). GABA and GABA receptors in the central nervous system and other organs. Int. Rev. Cytol..

[15] Qian X., Zhao X., Yu L., Yin Y., Zhang X.D., Wang L., Li J.X., Zhu Q., Luo J.L. (2023). Current status of GABA receptor subtypes in analgesia. Biomed. Pharmacother..

[16] Jembrek M.J., Vlainic J. (2015). GABA Receptors: Pharmacological Potential and Pitfalls. Curr. Pharm. Des..

[17] Ghit A., Assal D., Al-Shami A.S., Hussein D.E.E. (2021). GABA(A) receptors: structure, function, pharmacology, and related disorders. J. Genet. Eng. Biotechnol..

[18] Wang C., Hao H., He K., An Y., Pu Z., Gamper N., Zhang H., Du X. (2021). Neuropathic Injury-Induced Plasticity of GABAergic System in Peripheral Sensory Ganglia. Front. Pharmacol..

[19] Li C., Lei Y., Tian Y., Xu S., Shen X., Wu H., Bao S., Wang F. (2019). The etiological contribution of GABAergic plasticity to the pathogenesis of neuropathic pain. Mol. Pain.

[20] Yang S., Zhang B., Wang D., Hu S., Wang W., Liu C., Wu Z., Yang C. (2023). Role of GABAergic system in the comorbidity of pain and depression. Brain Res. Bull..

[21] Sousa E., Flames N. (2022). Transcriptional regulation of neuronal identity. Eur. J. Neurosci..

[22] Flitsch L.J., Laupman K.E., Brüstle O. (2020). Transcription Factor-Based Fate Specification and Forward Programming for Neural Regeneration. Front. Cell. Neurosci..

[23] Marmigere F., Carroll P. (2014). Neurotrophin signalling and transcription programmes interactions in the development of somatosensory neurons. Handb Exp. Pharmacol..

[24] Colasante G., Rubio A., Massimino L., Broccoli V. (2019). Direct Neuronal Reprogramming Reveals Unknown Functions for Known Transcription Factors. Front. Neurosci..

[25] Patodia S., Raivich G. (2012). Role of transcription factors in peripheral nerve regeneration. Front. Mol. Neurosci..

[26] Li M., Banton M.C., Min Q., Parkinson D.B., Dun X. (2021). Meta-Analysis Reveals Transcription Factor Upregulation in Cells of Injured Mouse Sciatic Nerve. Front. Cell. Neurosci..

[27] Nickerson D.S. (2014). Comment on Tesfaye et al. Mechanisms and management of diabetic painful distal symmetrical polyneuropathy. Diabetes Care.

[28] Boulton A.J.M., Vinik A.I., Arezzo J.C., Bril V., Feldman E.L., Freeman R., Malik R.A., Maser R.E., Sosenko J.M., Ziegler D., American Diabetes Association (2005). Diabetic neuropathies: a statement by the American Diabetes Association. Diabetes Care.

[29] Pham V.M., Matsumura S., Katano T., Funatsu N., Ito S. (2019). Diabetic neuropathy research: from mouse models to targets for treatment. Neural Regen. Res..

[30] Rosenberger D.C., Blechschmidt V., Timmerman H., Wolff A., Treede R.D. (2020). Challenges of neuropathic pain: focus on diabetic neuropathy. J. Neural Transm..

[31] Berta T., Qadri Y., Tan P.H., Ji R.R. (2017). Targeting dorsal root ganglia and primary sensory neurons for the treatment of chronic pain. Expert Opin. Ther. Targets.

[32] Yin Y., Yi M.H., Kim D.W. (2018). Impaired Autophagy of GABAergic Interneurons in Neuropathic Pain. Pain Res. Manag..

[33] Moore K.A., Kohno T., Karchewski L.A., Scholz J., Baba H., Woolf C.J. (2002). Partial peripheral nerve injury promotes a selective loss of GABAergic inhibition in the superficial dorsal horn of the spinal cord. J. Neurosci..

[34] Masocha W. (2015). Comprehensive analysis of the GABAergic system gene expression profile in the anterior cingulate cortex of mice with Paclitaxel-induced neuropathic pain. Gene Expr..

[35] Kanaani J., Kolibachuk J., Martinez H., Baekkeskov S. (2010). Two distinct mechanisms target GAD67 to vesicular pathways and presynaptic clusters. J. Cell Biol..

[36] Govindpani K., Calvo-Flores Guzman B., Vinnakota C., Waldvogel H.J., Faull R.L., Kwakowsky A. (2017). Towards a better understanding of GABAergic remodeling in Alzheimer’s disease. Int. J. Mol. Sci..

[37] Kuriyama K. (1994). Cerebral GABA receptors. Alcohol Alcohol.

[38] Plinkert P.K., Gitter A.H., Möhler H., Zenner H.P. (1993). Structure, pharmacology and function of GABA-A receptors in cochlear outer hair cells. Eur. Arch. Otorhinolaryngol..

[39] Ogawa N., Terashima T., Oka K., Chan L., Kojima H. (2018). Gene therapy for neuropathic pain using dorsal root ganglion-targeted helper-dependent adenoviral vectors with GAD67 expression. Pain Rep..

[40] Craner M.J., Klein J.P., Renganathan M., Black J.A., Waxman S.G. (2002). Changes of sodium channel expression in experimental painful diabetic neuropathy. Ann. Neurol..

[41] Hong S., Morrow T.J., Paulson P.E., Isom L.L., Wiley J.W. (2004). Early painful diabetic neuropathy is associated with differential changes in tetrodotoxin-sensitive and -resistant sodium channels in dorsal root ganglion neurons in the rat. J. Biol. Chem..

[42] Dib-Hajj S.D., Fjell J., Cummins T.R., Zheng Z., Fried K., LaMotte R., Black J.A., Waxman S.G. (1999). Plasticity of sodium channel expression in DRG neurons in the chronic constriction injury model of neuropathic pain. Pain.

[43] Hameed S. (2019). Na(v)1.7 and Na(v)1.8: Role in the pathophysiology of pain. Mol. Pain.

[44] Chattopadhyay M., Mata M., Fink D.J. (2008). Continuous delta-opioid receptor activation reduces neuronal voltage-gated sodium channel (NaV1.7) levels through activation of protein kinase C in painful diabetic neuropathy. J. Neurosci..

[45] Yu L., Yang F., Luo H., Liu F.Y., Han J.S., Xing G.G., Wan Y. (2008). The role of TRPV1 in different subtypes of dorsal root ganglion neurons in rat chronic inflammatory nociception induced by complete Freund's adjuvant. Mol. Pain.

[46] Cao E., Cordero-Morales J.F., Liu B., Qin F., Julius D. (2013). TRPV1 channels are intrinsically heat sensitive and negatively regulated by phosphoinositide lipids. Neuron.

[47] Walder R.Y., Radhakrishnan R., Loo L., Rasmussen L.A., Mohapatra D.P., Wilson S.P., Sluka K.A. (2012). TRPV1 is important for mechanical and heat sensitivity in uninjured animals and development of heat hypersensitivity after muscle inflammation. Pain.

[48] Shuba Y.M. (2020). Beyond Neuronal Heat Sensing: Diversity of TRPV1 Heat-Capsaicin Receptor-Channel Functions. Front. Cell. Neurosci..

[49] Mishra S.K., Tisel S.M., Orestes P., Bhangoo S.K., Hoon M.A. (2011). TRPV1-lineage neurons are required for thermal sensation. EMBO J..

[50] Allan D.W., Thor S. (2015). Transcriptional selectors, masters, and combinatorial codes: regulatory principles of neural subtype specification. Wiley Interdiscip. Rev. Dev. Biol..

[51] Belmonte-Mateos C., Pujades C. (2021). From Cell States to Cell Fates: How Cell Proliferation and Neuronal Differentiation Are Coordinated During Embryonic Development. Front. Neurosci..

[52] Sellers K., Zyka V., Lumsden A.G., Delogu A. (2014). Transcriptional control of GABAergic neuronal subtype identity in the thalamus. Neural Dev..

[53] Yang N., Chanda S., Marro S., Ng Y.H., Janas J.A., Haag D., Ang C.E., Tang Y., Flores Q., Mall M. (2017). Generation of pure GABAergic neurons by transcription factor programming. Nat. Methods.

[54] Liu Y.H., Tsai J.W., Chen J.L., Yang W.S., Chang P.C., Cheng P.L., Turner D.L., Yanagawa Y., Wang T.W., Yu J.Y. (2017). Ascl1 promotes tangential migration and confines migratory routes by induction of Ephb2 in the telencephalon. Sci. Rep..

[55] Raina A., Mahajani S., Bähr M., Kügler S. (2020). Neuronal Trans-differentiation by Transcription Factors Ascl1 and Nurr1: Induction of a Dopaminergic Neurotransmitter Phenotype in Cortical GABAergic Neurons. Mol. Neurobiol..

[56] Yuan F., Fang K.H., Hong Y., Xu S.B., Xu M., Pan Y., Liu Y. (2020). LHX6 is essential for the migration of human pluripotent stem cell-derived GABAergic interneurons. Protein Cell.

[57] Le T.N., Zhou Q.P., Cobos I., Zhang S., Zagozewski J., Japoni S., Vriend J., Parkinson T., Du G., Rubenstein J.L., Eisenstat D.D. (2017). GABAergic Interneuron Differentiation in the Basal Forebrain Is Mediated through Direct Regulation of Glutamic Acid Decarboxylase Isoforms by Dlx Homeobox Transcription Factors. J. Neurosci..

[58] Anderson S.A., Eisenstat D.D., Shi L., Rubenstein J.L. (1997). Interneuron migration from basal forebrain to neocortex: dependence on Dlx genes. Science.

[59] Sandberg M., Flandin P., Silberberg S., Su-Feher L., Price J.D., Hu J.S., Kim C., Visel A., Nord A.S., Rubenstein J.L.R. (2016). Transcriptional Networks Controlled by NKX2-1 in the Development of Forebrain GABAergic Neurons. Neuron.

[60] Traxler L., Edenhofer F., Mertens J. (2019). Next-generation disease modeling with direct conversion: a new path to old neurons. FEBS Lett..

[61] Iwafuchi-Doi M., Zaret K.S. (2014). Pioneer transcription factors in cell reprogramming. Genes Dev..

[62] Pang Z.P., Yang N., Vierbuchen T., Ostermeier A., Fuentes D.R., Yang T.Q., Citri A., Sebastiano V., Marro S., Südhof T.C., Wernig M. (2011). Induction of human neuronal cells by defined transcription factors. Nature.

[63] Parkinson L.M., Gillen S.L., Woods L.M., Chaytor L., Marcos D., Ali F.R., Carroll J.S., Philpott A. (2022). The proneural transcription factor ASCL1 regulates cell proliferation and primes for differentiation in neuroblastoma. Front. Cell Dev. Biol..

[64] Riva C., Hajduskova M., Gally C., Suman S.K., Ahier A., Jarriault S. (2022). A natural transdifferentiation event involving mitosis is empowered by integrating signaling inputs with conserved plasticity factors. Cell Rep..

[65] Masserdotti G., Gascón S., Götz M. (2016). Direct neuronal reprogramming: learning from and for development. Development.

[66] Eade K.T., Fancher H.A., Ridyard M.S., Allan D.W. (2012). Developmental transcriptional networks are required to maintain neuronal subtype identity in the mature nervous system. PLoS Genet..

[67] Black J.B., McCutcheon S.R., Dube S., Barrera A., Klann T.S., Rice G.A., Adkar S.S., Soderling S.H., Reddy T.E., Gersbach C.A. (2020). Master Regulators and Cofactors of Human Neuronal Cell Fate Specification Identified by CRISPR Gene Activation Screens. Cell Rep..

[68] Stanslowsky N., Haase A., Martin U., Naujock M., Leffler A., Dengler R., Wegner F. (2014). Functional differentiation of midbrain neurons from human cord blood-derived induced pluripotent stem cells. Stem Cell Res. Ther..

[69] Gutierrez R. (2016). The plastic neurotransmitter phenotype of the hippocampal granule cells and of the moss in their messy fibers. J. Chem. Neuroanat..

[70] Li Y.F., Jackson K.L., Stern J.E., Rabeler B., Patel K.P. (2006). Interaction between glutamate and GABA systems in the integration of sympathetic outflow by the paraventricular nucleus of the hypothalamus. Am. J. Physiol. Heart Circ. Physiol..

[71] Sears S.M., Hewett S.J. (2021). Influence of glutamate and GABA transport on brain excitatory/inhibitory balance. Exp. Biol. Med..

[72] Kung L.H., Gong K., Adedoyin M., Ng J., Bhargava A., Ohara P.T., Jasmin L. (2013). Evidence for glutamate as a neuroglial transmitter within sensory ganglia. PLoS One.

[73] Malet M., Brumovsky P.R. (2015). VGLUTs and Glutamate Synthesis-Focus on DRG Neurons and Pain. Biomolecules.

[74] Gazon H., Barbeau B., Mesnard J.M., Peloponese J.M. (2017). Hijacking of the AP-1 Signaling Pathway during Development of ATL. Front. Microbiol..

[75] Luis-Delgado O.E., Barrot M., Rodeau J.L., Ulery P.G., Freund-Mercier M.J., Lasbennes F. (2006). The transcription factor DeltaFosB is recruited by inflammatory pain. J. Neurochem..

[76] Zhu X., Li F., Wang M., Su H., Wu X., Qiu H., Zhou W., Shan C., Wang C., Wei L. (2021). Integrated Analysis of Omics Data Reveal AP-1 as a Potential Regulation Hub in the Inflammation-Induced Hyperalgesia Rat Model. Front. Immunol..

[77] Gillardon F., Wiesner R.J., Zimmermann M. (1992). Expression of the junD proto-oncogene in the rat spinal cord and skin following noxious cutaneous ultraviolet irradiation. Neurosci. Lett..

[78] Hu Y.F., Li R. (2002). JunB potentiates function of BRCA1 activation domain 1 (AD1) through a coiled-coil-mediated interaction. Genes Dev..

[79] Ubeda M., Vallejo M., Habener J.F. (1999). CHOP enhancement of gene transcription by interactions with Jun/Fos AP-1 complex proteins. Mol. Cell. Biol..

[80] Agarwal S.K., Guru S.C., Heppner C., Erdos M.R., Collins R.M., Park S.Y., Saggar S., Chandrasekharappa S.C., Collins F.S., Spiegel A.M. (1999). Menin interacts with the AP1 transcription factor JunD and represses JunD-activated transcription. Cell.

[81] Chu H.M., Tan Y., Kobierski L.A., Balsam L.B., Comb M.J. (1994). Activating transcription factor-3 stimulates 3',5'-cyclic adenosine monophosphate-dependent gene expression. Mol. Endocrinol..

[82] Nestler E.J. (2015). ΔFosB: a transcriptional regulator of stress and antidepressant responses. Eur. J. Pharmacol..

[83] Milde-Langosch K. (2005). The Fos family of transcription factors and their role in tumourigenesis. Eur. J. Cancer.

[84] Nomaru H., Sakumi K., Katogi A., Ohnishi Y.N., Kajitani K., Tsuchimoto D., Nestler E.J., Nakabeppu Y. (2014). Fosb gene products contribute to excitotoxic microglial activation by regulating the expression of complement C5a receptors in microglia. Glia.

[85] Gao Y.J., Ji R.R. (2009). c-Fos and pERK, which is a better marker for neuronal activation and central sensitization after noxious stimulation and tissue injury?. Open Pain J..

[86] Bando S.Y., Silva F.N., Costa L.d.F., Silva A.V., Pimentel-Silva L.R., Castro L.H., Wen H.T., Amaro E., Moreira-Filho C.A. (2013). Complex network analysis of CA3 transcriptome reveals pathogenic and compensatory pathways in refractory temporal lobe epilepsy. PLoS One.

[87] Samaan G., Yugo D., Rajagopalan S., Wall J., Donnell R., Goldowitz D., Gopalakrishnan R., Venkatachalam S. (2010). Foxn3 is essential for craniofacial development in mice and a putative candidate involved in human congenital craniofacial defects. Biochem. Biophys. Res. Commun..

[88] Zhu X., Huang B., Zhao F., Lian J., He L., Zhang Y., Ji L., Zhang J., Yan X., Zeng T. (2023). p38-mediated FOXN3 phosphorylation modulates lung inflammation and injury through the NF-kappaB signaling pathway. Nucleic Acids Res..

[89] Ji H., Kim K.R., Park J.J., Lee J.Y., Sim Y., Choi H., Kim S. (2023). Combination Gene Delivery Reduces Spinal Cord Pathology in Rats With Peripheral Neuropathic Pain. J. Pain.

[90] Lee J.Y., Choi H.Y., Park C.S., Pyo M.K., Yune T.Y., Kim G.W., Chung S.H. (2019). GS-KG9 ameliorates diabetic neuropathic pain induced by streptozotocin in rats. J. Ginseng Res..

[91] Ni C.M., Sun H.P., Xu X., Ling B.Y., Jin H., Zhang Y.Q., Zhao Z.Q., Cao H., Xu L. (2020). Spinal P2X7R contributes to streptozotocin-induced mechanical allodynia in mice. J. Zhejiang Univ. Sci. B.

[92] Yu H., Fischer G., Hogan Q.H. (2016). AAV-Mediated Gene Transfer to Dorsal Root Ganglion. Methods Mol. Biol..

[93] Jan A., Richner M., Vaegter C.B., Nyengaard J.R., Jensen P.H. (2019). Gene Transfer in Rodent Nervous Tissue Following Hindlimb Intramuscular Delivery of Recombinant Adeno-Associated Virus Serotypes AAV2/6, AAV2/8, and AAV2/9. Neurosci. Insights.

[94] Islam A., Tom V.J. (2022). The use of viral vectors to promote repair after spinal cord injury. Exp. Neurol..

[95] Hwang S.M., Rahman M.M., Go E.J., Kim Y.H., Park C.K. (2024). Specific transcription factors Ascl1 and Lhx6 attenuate diabetic neuropathic pain by modulating spinal neuroinflammation and microglial activation in mice. Biomed. Pharmacother..

[96] Rahman M.M., Jo H.J., Park C.K., Kim Y.H. (2022). Diosgenin Exerts Analgesic Effects by Antagonizing the Selective Inhibition of Transient Receptor Potential Vanilloid 1 in a Mouse Model of Neuropathic Pain. Int. J. Mol. Sci..

[97] Rahman M.M., Lee J.Y., Kim Y.H., Park C.K. (2023). Epidural and Intrathecal Drug Delivery in Rats and Mice for Experimental Research: Fundamental Concepts, Techniques, Precaution, and Application. Biomedicines.

