# Supplementary material for: Modulation of pain sensitivity by Ascl1- and Lhx6-dependent GABAergic neuronal function in streptozotocin diabetic mice
Source: Mol Ther. 2024 Dec 30;33(2):786–804. doi: 10.1016/j.ymthe.2024.12.039 (PMC11852955; doi:10.1016/j.ymthe.2024.12.039)
Supplement: Document S1. Figures S1–S7 and Tables S1–S3 [file mmc1.pdf]

YMTHE, Volume *33*

Supplemental Information

Modulation of pain sensitivity by *Ascl1*- and *Lhx6*-dependent GABAergic neuronal function in streptozotocin diabetic mice

Sung-Min Hwang, Md. Mahbubur Rahman, Eun Jin Go, Jueun Roh, Rayoung Park, Sung-Gwon Lee, Minyeop Nahm, Temugin Berta, Yong Ho Kim, and Chul-Kyu Park

Supplemental information

Table S1. Effects of *Ascl1* and *Lhx6* on the final body weight and food and water intake of normal and diabetic mice at 42 days

	**NC**	**STZ**	***Ascl1***	***LhX6***	***Ascl1 + LhX6***
Initial BW	23.02 ± 0.33	22.87 ± 0.47	23.62 ± 0.33	23.45 ± 0.50	23.38 ± 0.46
Final BW	30.79 ± 1.06	19.01 ± 0.51[a]	19.68 ± 0.33[a]	19.56 ± 0.32[a]	19.74 ± 0.36[a]
Water intake (ml/day/mouse)	4.11 ± 0.05	18.94 ± 1.73[a]	18.59 ± 1.60[a]	19.03 ± 1.70[a]	18.02 ± 1.52[a]
Feed intake (g/day/mouse)	3.53 ± 0.06	6.98 ± 0.12[a]	6.96 ± 0.13[a]	6.96 ± 0.16[a]	6.93 ± 0.14[a]

NC, normal control mice treated with lentiviral vectors; STZ, diabetic control mice treated with lentiviral vectors; *Ascl1*, *Ascl1* transfected lentiviral treated diabetic mice; *LhX6*, *LhX6* transfected lentiviral treated diabetic mice; *Ascl1* + *LhX6*, *Ascl1* and *LhX6* transfected lentiviral treated diabetic mice; BW, body weight. Data are reported as mean ± standard error of the mean (n = 5). [a]$p < 0.001$, Bonferroni post-hoc test following one-way analysis of variance versus the NC group. No significant difference was observed between the STZ group and other diabetic treated groups.

Table S2. Effects of *Ascl1* and *Lhx6* on the final body weight and food and water intake of normal mice at 28 days

	**NC**	***Ascl1***	***LhX6***	***Ascl1 + LhX6***
Initial BW	23.00 ± 0.32	22.74 ± 0.52	23.24 ± 0.41	23.00 ± 0.33
Final BW	29.31 ± 0.63	28.68 ± 0.74	28.92 ± 0.69	28.55 ± 0.66
Water intake (ml/day/mouse)	4.34 ± 0.06	4.34 ± 0.08	4.30 ± 0.04	4.32 ± 0.06
Feed intake (g/day/mouse)	3.35 ± 0.14	3.34 ± 0.15	3.36 ± 0.14	3.33 ± 0.13

NC, normal control mice treated with lentiviral vectors; *Ascl1*, *Ascl1* transfected lentiviral treated normal mice; *LhX6*, *LhX6* transfected lentiviral treated normal mice; *Ascl1* + *LhX6*, *Ascl1* and *LhX6* transfected lentiviral treated normal mice; BW, body weight. Data are reported as mean ± standard error of the mean (n = 5).

Table S3. Primers used for quantitative real-time polymerase chain reaction amplification

Gene abbreviation	Forward primer sequence(5’-3’)	Reverse primer sequence(5’-3’)
GAD65	GCTGGAACCACCGTGTATGG	TCCACGTGCATCCAGATCTTAT
GAD67	TCCACCATCAACGGCATTAA	AGCGGCAGGTGTTGGATAAC
vGAT	TCATCGAGCTGGTGATGACG	CTTGGACACGGCCTTGAGAT
GABAB1	ACGTCACCTCGGAAGGTTG	CACAGGCAGGAAATTGATGGC
GABAB2	CAGCAAGCGTTCGGGTGTA	GTCTTGGCGATGACCCAGAT
GABA-T	GGGGTCATGGCCTTCTTGTT	AGTGGTCCATCATAATCAAATTCAA
GABAAR-α1	GCCAGAAATTCCCTCCCGAA	CATCCCACGCATACCCTCTC
GABAAR-α2	GCTCGAAATTCCCTCCCGAA	ACTACACTCTTCCCGTCCCA
GABAAR-α4	CTCAGACGGAAGATGGGCTAC	ATGCTTAGGGTGGTCATCGTG
GABAAR-α5	ATGAAACTGCGGAGAGAGCC	CAGTCATTGCCTCCCATCGT
GABAAR-β1	CTACGGATCACAACCACTGC	CTCCCTCTCCTCCATTCCAG
GABAAR-β2	AAGATGCGCCTGGATGTCAA	TGCGGCCAAAACTATGCCTA
GABAAR-β3	TTGACGCCTTGATGACAGCC	TTGACGCCTTGATGACAGCC
GABAAR-ɣ1	GCAGCACTCATGGAATACGG	ATCATCTTCCCCTTGTGGCA
GABAAR-ɣ2	TATCGCTCTACCCAGGCTTC	TGTCATACCCTTCCAGCAGG
GABAAR-ɣ3	GATGCGACACCAGCAAGAAC	CAGTGCGGCAAAGACAAACA
GABAAR-δ	TACCAACGAGACCTTAGGCC	GTCACATCATGGAACCAGGC
TRPV1	GAGCAAGAACATCTGGAAG	GTGTTCCAGGTAGTCCAGTT
TRPA1	TCCTATACTGGAAGCAGCGA	CTCCTGATTGCCATCGACT
Piezo1	GACGCCTCACAAGGA AAGC	GGGCAGCATCTATGTCATCC
Nav1.7	TCGTACCCCATAGACCCCG	CTGATTAGTCGTGCCGCTG
Nav1.8	GTGGTCACCATGGCGTATGAAG	CTGGAGCGAGGTCGTGTCAA
Ascl1	CAACCGGGTCAAGTTGGTCA	AGTAGGACGAGACCGGAGAA
Lhx6	GAGAGTCAGGTACAGTGCGG	GAGAGCGGCCCATCCATATC
Dlx1	CAGTTGCAGGCTTTGAACC	ACTTGGAGCGTTTGTTCTGG
Dlx2	GCCTCACCCAAACTCAGG	GCCGCTTTTCCACATCTTC
Dlx5	CGACTTCCAAGCTCCGTTC	TTCTTTCTCTGGCTGGCTG
Nkx2.1	TGTCCTCGGAAAGACAGCAT	TCCTCCAGGGGACTCAAGAT
Nkx2.2	GCGGAGAAAGGTATGGAGGT	AGGTCCTGGGCTTTGAGC
TNF-α	CGTCAGCCGATTTGCTATCT	CGGACTCCGCAAAGTCTAAG
IL-1β	TGACGGACCCCAAAAGATGA	TCTCCACAGCCACAATGAGT
IL-6	AGACTTCCATCCAGTTGCCT	AGTGCATCATCGTTGTTCATACA
IL-4	CATGGGAAAACTCCATGCTT	TGGACTCATTCATGGTGCAG
IL-10	GCTCCTAGAGCTGCGGACT	TCATTTCCGATAAGGCTTGG
IL-13	TCCAATTGCAATGCCATCTA	TGGGCTACTTCGATTTTGGT
vGlut1	GGTGGAGGGGGTCACATAC	AGATCCCGAAGCTGCCATAGA
vGlut2	CCCTGGAGGTGCCTGAGAA	GCGGTGGATAGTGCTGTTGTT
Tbr1	CCGGAGACTCAGTTCATCGC	GCCCGTGTAGATCGTGTCAT
Tbr2	GTGACGGCCTACCAAAACAC	CCACCTCTTCGCTGAATCGT
NeuroD1	GGTGCCTTGCTATTCTAAGACGC	GCAAAGCGTCTGAACGAAGGAG
NeuroG2	ATCCGAGCAGCACTAACACG	GCTGAGGCACAGTTAGAGCC
GAPDH	CAA TGC ATCCTGCACCACCAA	GTCATTGAGAGCA ATGCCAGC

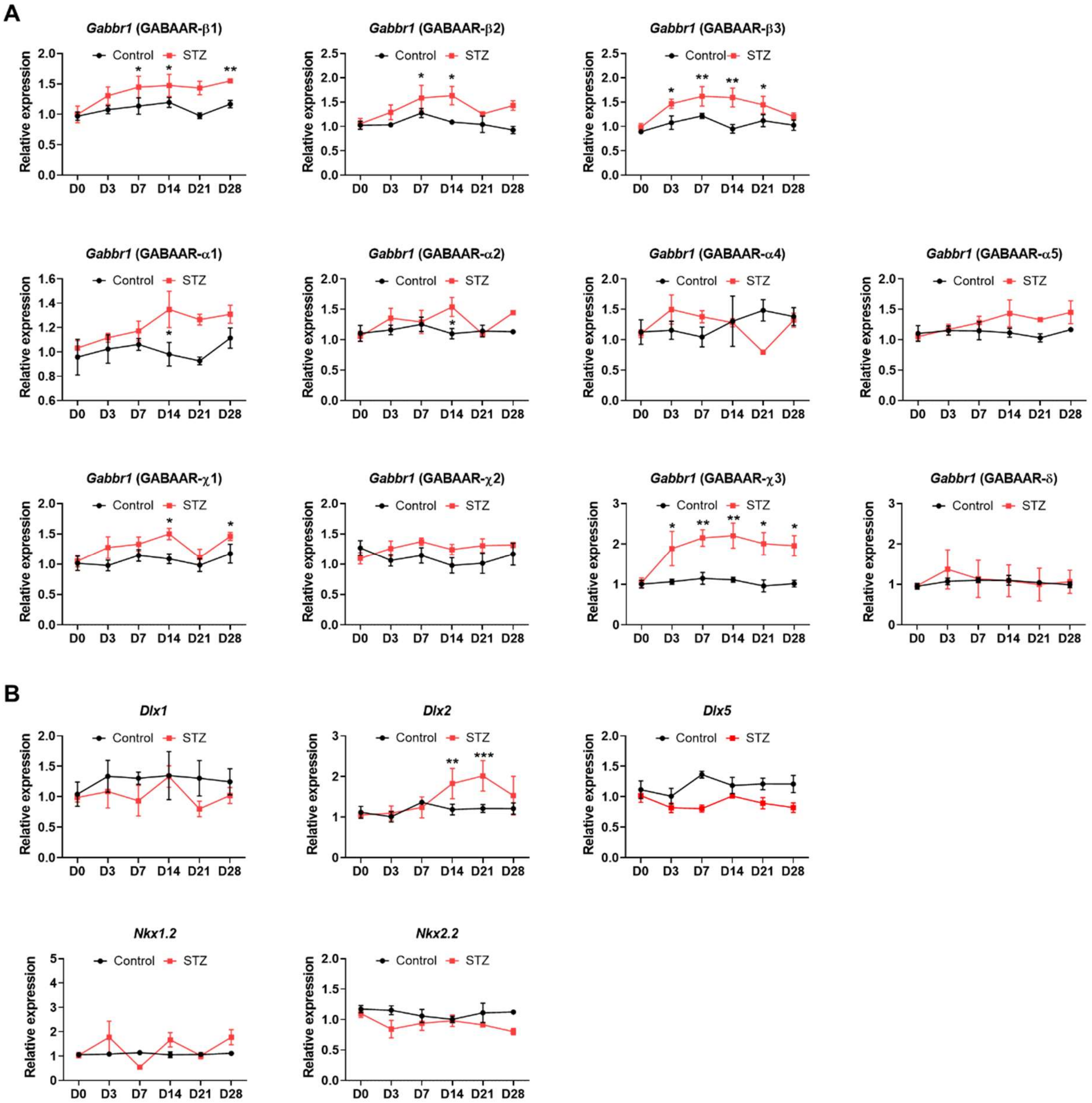

Figure S1. GABAergic neuron-specific genes and their transcription factors in the DRG of mice with diabetes. (A) Relative mRNA expression levels of GABAergic neuron-specific genes. (B) Relative mRNA expression levels of transcription factors in controlling the GABAergic neuron subtype identity. Data are reported as mean ± standard error of the mean (n = 5). *$p < 0.05$, **$p < 0.01$, ***$p < 0.001$, Dunnett's multiple comparisons tests by one-way analysis of variance versus day 0. STZ, streptozotocin; DRG, dorsal root ganglia; GABA, gamma-aminobutyric acid.

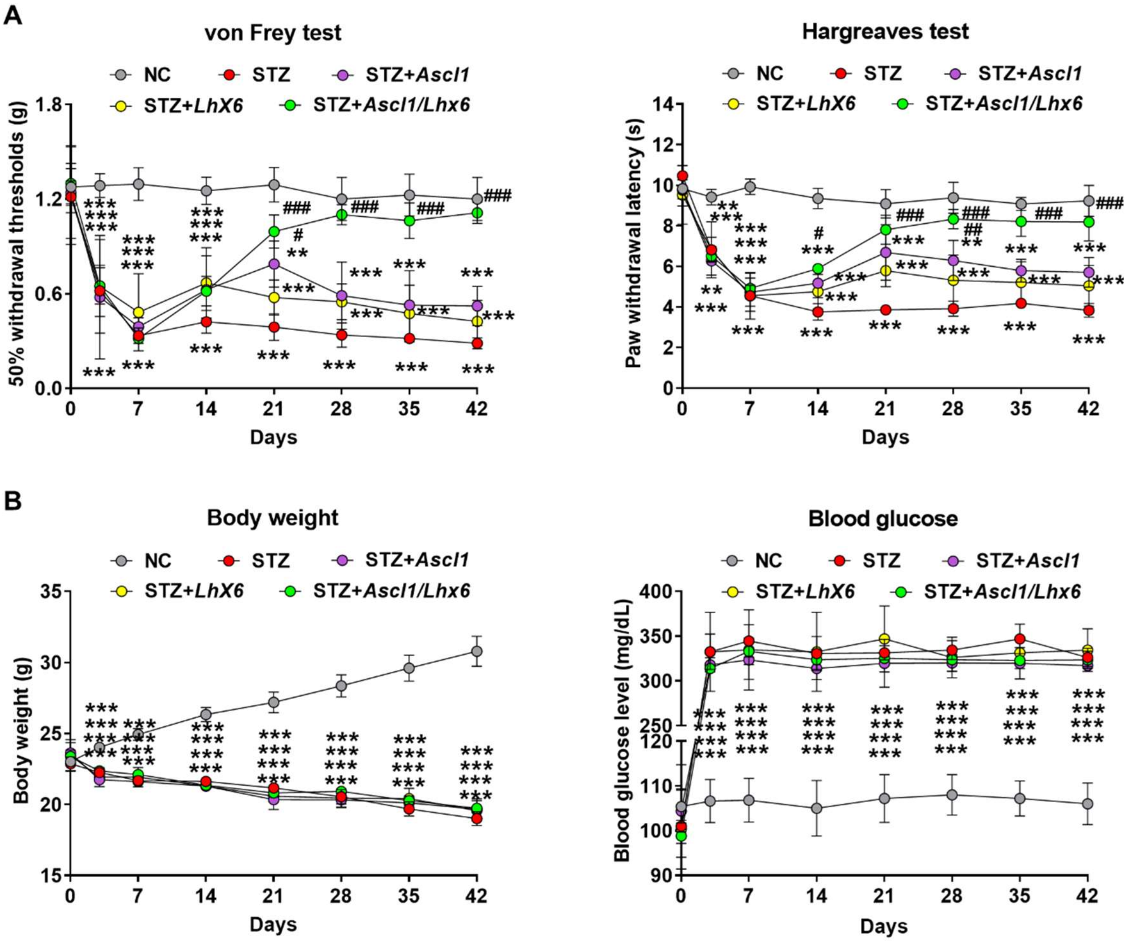

Figure S2. Effects of *Ascl1* and *Lhx6* on pain sensitivity, body weight, and blood glucose levels in diabetic mice over 42 days. (A) Mechanical and thermal pain sensitivity test results demonstrating the inhibitory effects of lentiviral-mediated *Ascl1* and *Lhx6* expression in STZ-induced diabetic mice. (B) Body weight (BW) and blood glucose levels measured in diabetic mice treated with lentiviral vectors. Experimental groups include the NC (normal control mice treated with lentiviral vectors), STZ (diabetic control mice treated with lentiviral vectors), *Ascl1* (*Ascl1*-transfected lentiviral-treated diabetic mice), *Lhx6* (*Lhx6*-transfected lentiviral-treated diabetic mice), and *Ascl1* + *Lhx6* (combined *Ascl1* and *Lhx6* lentiviral-treated diabetic mice) groups. Data are expressed as mean ± standard error of the mean (n = 5). $^{***}p < 0.001$, Bonferroni post hoc test following one-way ANOVA versus the NC group; $^{\#}p < 0.05$, $^{\#\#}p < 0.01$, and $^{\#\#\#}p < 0.001$, Bonferroni post hoc test following one-way ANOVA versus the diabetic control group. ANOVA, analysis of variance; STZ, streptozotocin.

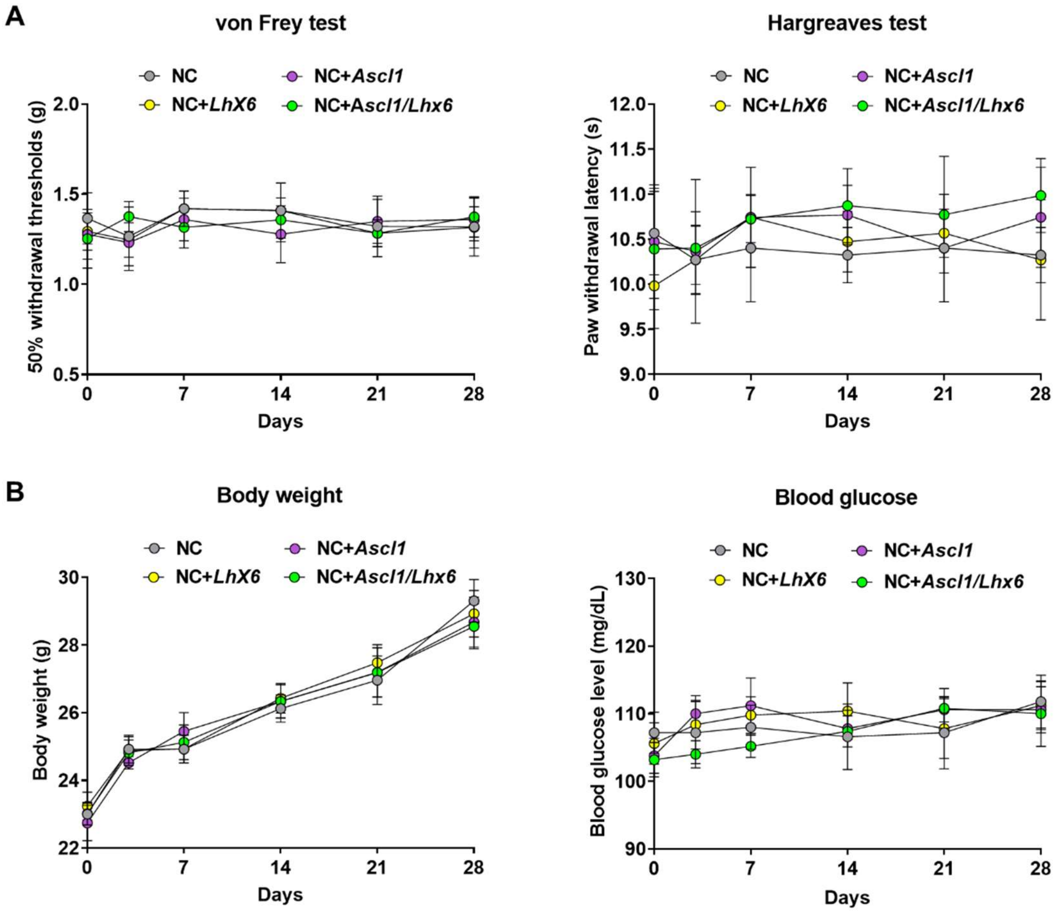

Figure S3. Effects of *Ascl1* and *Lhx6* on pain sensitivity, body weight, and blood glucose levels in normal mice over 28 days. (A) Mechanical and thermal pain sensitivity test results demonstrating the effects of lentiviral-mediated *Ascl1* and *Lhx6* expression on normal mice. (B) Body weight (BW) and blood glucose levels in normal mice treated with lentiviral vectors. Experimental groups include the NC (normal control mice treated with lentiviral vectors), *Ascl1* (*Ascl1*-transfected lentiviral-treated normal mice), *Lhx6* (*Lhx6*-transfected lentiviral-treated normal mice), and *Ascl1* + *Lhx6* (combined *Ascl1* and *Lhx6* lentiviral-treated normal mice) groups. Data are expressed as mean ± standard error of the mean (n = 5).

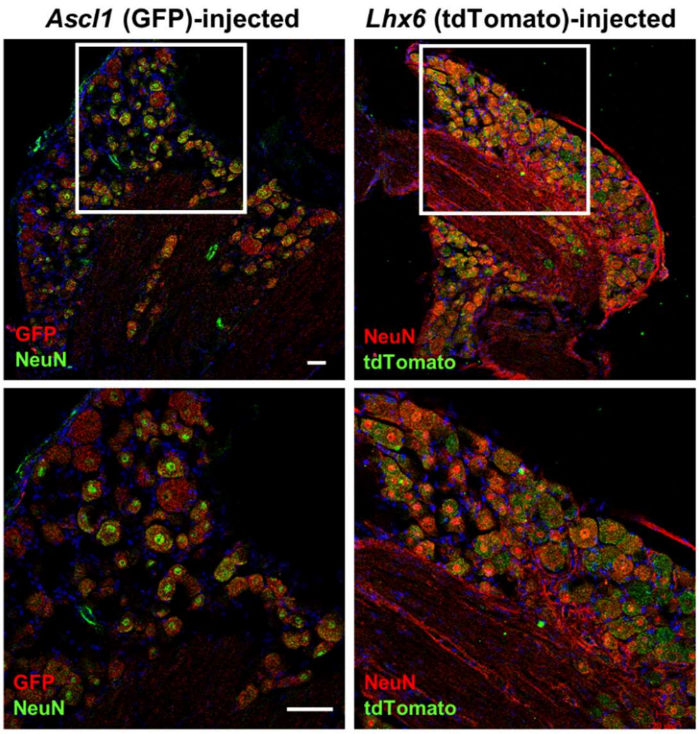

Figure S4. Co-localization of NeuN with GFP (reporter for *Ascl1*) and tdTomato (reporter for *Lhx6*) in DRG tissues following intrathecal lentiviral vector administration. Representative immunohistochemistry images showing GFP (green) and tdTomato (red) fluorescence in dorsal root ganglia (DRG) tissues, co-labeled with the neuronal marker NeuN (red for GFP images and green for tdTomato images). The upper images were taken using a 10X objective, while the lower images were taken using a 20X objective. DRG tissues were harvested four weeks after intrathecal injection of Lenti-hSyn-mAscl-P2A-EGFP (GFP) or Lenti-hSyn-mLhx6-P2A-dTomato (tdTomato) lentiviral vectors. Co-localization of fluorescence signals with NeuN confirms neuronal expression of GFP and tdTomato. Scale bar = 20 μm.

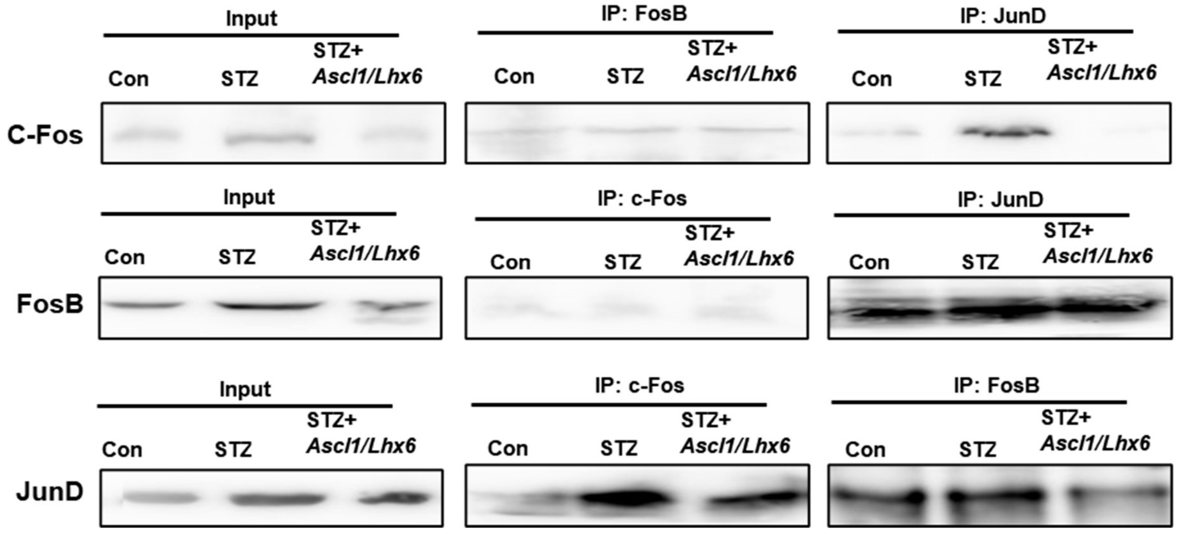

Figure S5. The interactions of JunD, FosB, and C-fos were detected in three groups using western blotting. DRG was lysed, and the resulting samples were subjected to co-immunoprecipitation using antibodies against c-fos, fosB, and JunD. Following this, immunoblotting was performed with the same antibodies to detect the interaction among these proteins. DRG, dorsal root ganglia; STZ, streptozotocin.

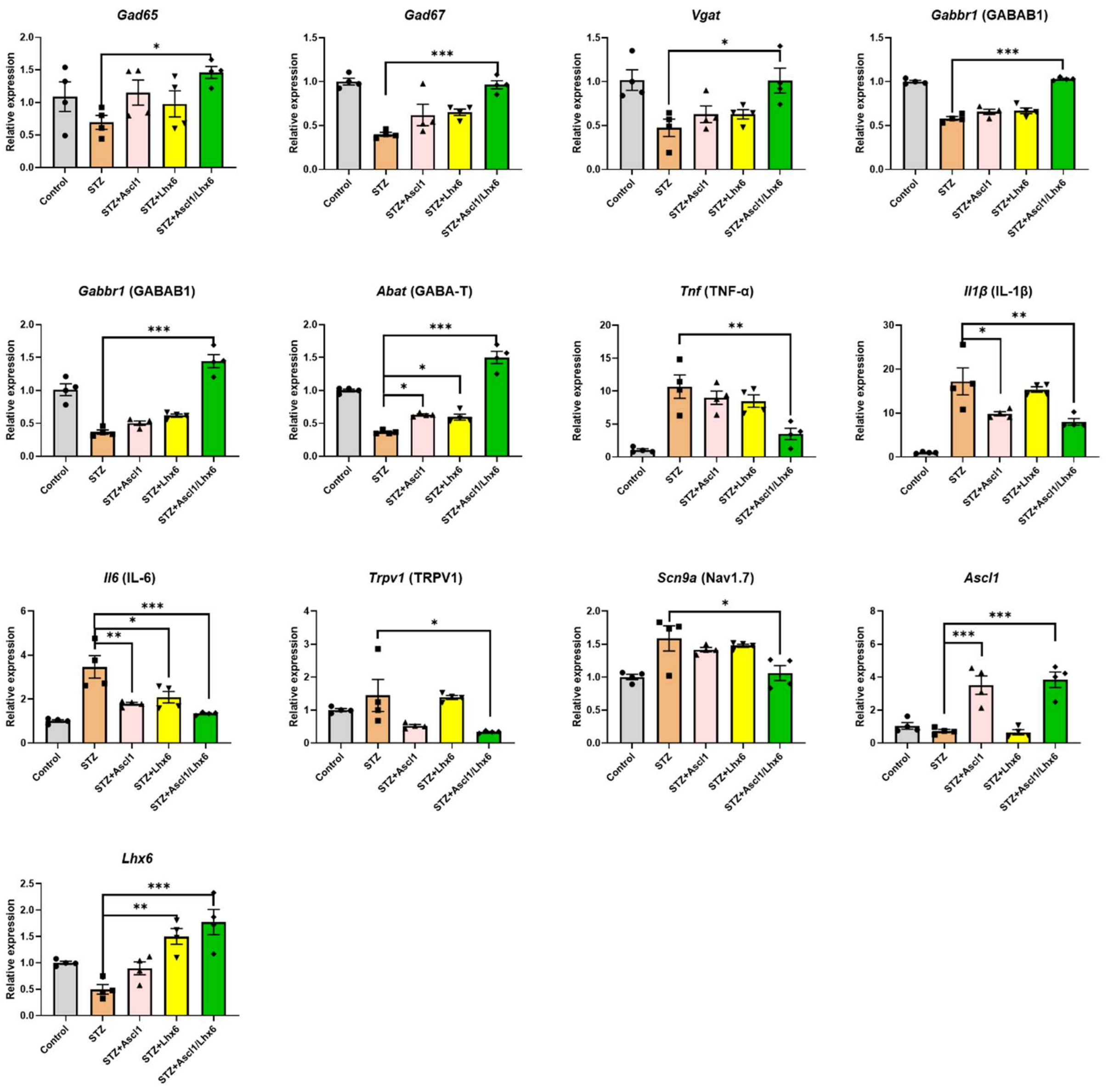

Figure S6. Regulatory functions of Ascl1 and/or Lhx6 on specific genes in the DRG of diabetic mice. Relative mRNA expression level of GABA-related genes (*Gad65, Gad 67, Vgat, Gabra1, Gabra2, Abat*), pro-inflammatory genes (*Tnf, Il-1β*, *Il-6*), pain-related channels (*Trpv1* and *Scn9a*), and transcription factors (*Ascl1* and *Lhx6*) were assessed. β-actin was used as an internal control. Data are reported as mean ± standard error of the mean (n = 5). *$p < 0.05$, **$p < 0.01$, ***$p < 0.001$, Tukey's multiple comparisons tests by two-way analysis of variance versus the STZ-treated group. TNF, tumor necrosis factor; IL, interleukin; GABA, gamma-aminobutyric acid; DRG, dorsal root ganglia; STZ, streptozotocin.

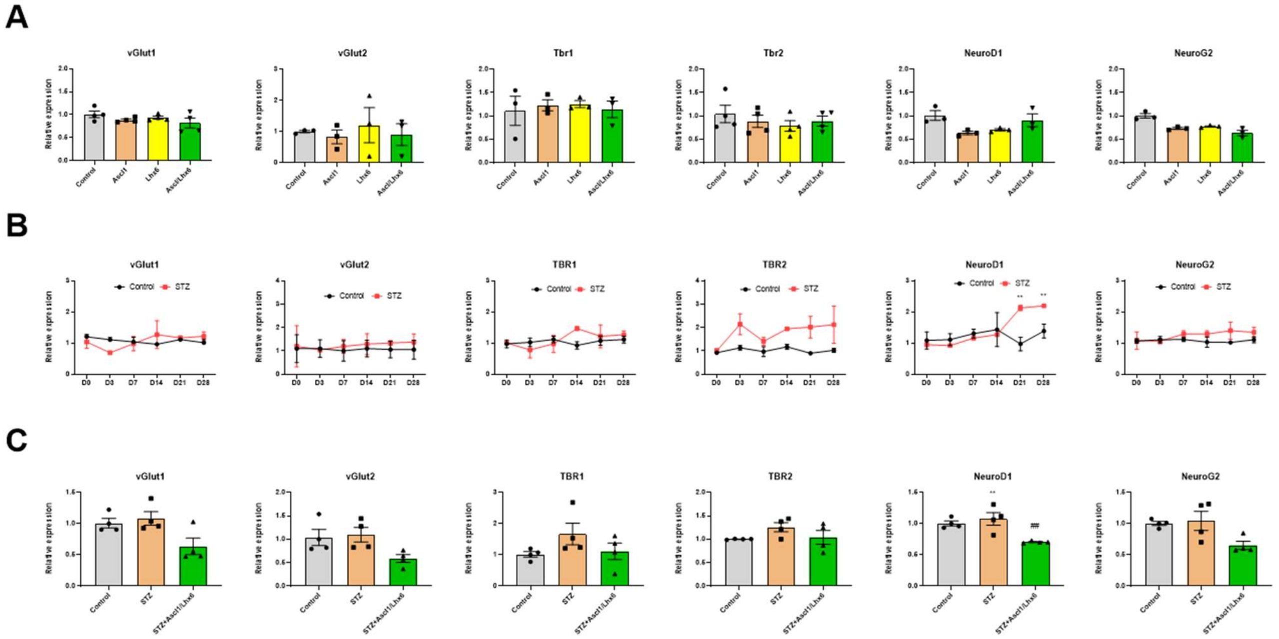

Figure S7. Glutamatergic neuron-specific genes and their transcription factors in the DRG of diabetic mice. (A) Relative mRNA expression levels of glutamatergic neuron-specific genes and transcription factors controlling the glutamatergic neuron subtype identity in primary DRG neurons after treatment with *Ascl1* and *Lhx6*. (B) Relative mRNA expression levels of glutamatergic neuron-specific genes and transcription factors controlling the glutamatergic neuron subtype identity in STZ mice. (C) Relative mRNA expression levels of glutamatergic neuron-specific genes and transcription factors controlling the glutamatergic neuron subtype identity in three groups. $^{**}p < 0.01$, Dunnett's multiple comparisons test by one-way analysis of variance versus day 0. $^{\#\#}p < 0.01$, versus the streptozotocin-diabetic control group. STZ, streptozotocin; DRG, dorsal root ganglia.
